# Optimization of microwave-assisted extraction of antioxidant compounds from spring onion leaves using Box–Behnken design

**DOI:** 10.1038/s41598-023-42303-x

**Published:** 2023-09-10

**Authors:** Giovanna Aquino, Manuela Giovanna Basilicata, Carlo Crescenzi, Vincenzo Vestuto, Emanuela Salviati, Michele Cerrato, Tania Ciaglia, Francesca Sansone, Giacomo Pepe, Pietro Campiglia

**Affiliations:** 1 Department of Pharmacy, University of Salerno, 84084 Fisciano, SA Italy; 2 PhD Program in Drug Discovery and Development, University of Salerno, Fisciano, SA Italy

**Keywords:** Plant sciences, Chemistry

## Abstract

Many studies have explored the extraction of bioactive compounds from different onion solid wastes, such as bulb, skin, and peel. However, onion leaves have received limited attention despite their potential as a valuable source of nutraceutical compounds. This study aimed to valorise, for the first time, the agricultural waste in the form of spring onion leaves (CN, *Cipollotto Nocerino*) to obtain antioxidant-rich polyphenolic extracts. A Box–Behnken design (BBD) was used to assess the impact of microwave-assisted extraction (MAE) variables (temperature, time, extraction volume, and ethanol concentration) on total polyphenol content (TPC) measured by Folin–Ciocalteu method and the antioxidant power determined by FRAP assay. Response surface methodology (RSM) was applied, and regression equations, analysis of variance, and 3D response curves were developed. Our results highlighted that the TPC values range from 0.76 to 1.43 mg GAE g^−1^ dw, while the FRAP values range from 8.25 to 14.80 mmol Fe(II)E g^−1^ dw. The optimal extraction conditions predicted by the model were 60 °C, 22 min, ethanol concentration 51% (v/v), and solvent volume 11 mL. These conditions resulted in TPC and FRAP values of 1.35 mg GAE g^−1^ dw and 14.02 mmol Fe(II)E g^−1^ dw, respectively. Furthermore, the extract obtained under optimized conditions was characterized by UHPLC-ESI-Orbitrap-MS analysis. LC/MS–MS platform allowed us to tentatively identify various compounds belonging to the class of flavonoids, saponins, fatty acids, and lipids. Finally, the ability of CN optimal extract to inhibit the intracellular reactive oxygen species (ROS) release in a hepatocarcinoma cell line using an H_2_O_2_-induced oxidative stress model, was evaluated. The results highlighted the potential of CN extract as a valuable source of polyphenols with significant antioxidant properties, suitable for various applications in the food and pharmaceutical industries.

## Introduction

The agri-food sector generates a substantial amount of waste, including crop residues like stalks, leaves, and husks, as well as by-products from food processing, ranging from peels and shells to stems, expired or unsold food, and packaging materials. Effectively managing and reducing this waste is crucial for promoting environmental sustainability, enhancing resource efficiency, and preventing food loss and waste^1,2^.

Implementing proper waste management practices plays a vital role in minimizing the sector’s environmental impact. Strategies such as recycling, composting, and optimizing packaging can significantly reduce waste generation^3^. Recycling and reusing packaging materials not only conserve resources but also reduce the volume of waste that ends up in landfills. Composting organic waste, such as crop residues and food processing by-products, helps produce nutrient-rich soil amendments and reduces reliance on synthetic fertilizers^4–6^.

Preventing food loss and waste is a fundamental aspect of building a more sustainable food system. By reducing food waste at various stages of the supply chain, valuable resources like water, energy, and land can be conserved, while also minimizing associated greenhouse gas emissions^7^.

Onion (*Allium cepa* L.) is an example of a widely consumed vegetable that contributes considerably to municipal and industrial wastes, consisting of onion skins, outer fleshy scales, roots, leaves and the apical and basal trimming of bulbs and are commonly known as onion solid wastes (OSW)^8,9^.

An enormous amount of OSW is generated in several countries. For example, in California, USA, approximately 100,000 tons of OSW are produced annually. Similarly, in the European Union, particularly in Spain, Holland, and the UK, about 500,000 tons of OSW are generated each year^10^. Despite being considered waste products, OSW are of great interest for the recovery of active ingredients. Several studies have contributed to the development and validation of extractive techniques designed for the isolation and purification of bioactive compounds from onion bulb^11,12^, skin^13–16^, peel^17,18^ and solid wastes^19,20^. However, despite their significant nutraceutical potential, research focused on onion leaves has remained limited. Fresh onion leaves contain high levels of bioactive compounds such as polyphenols, flavonoids, carotenoids, vitamins, and chlorophylls^21,22^. This study aimed to investigate, for the first time, the antioxidant properties of green onion stalks sourced from the “*Cipollotto Nocerino*” onion (CN) variety. These leaves are characterized by intensely green colour, linear in shape, and end in a pointed tip. They constitute the primary by-product of CN, measuring approximately 15–30 cm in length, a size six to seven times larger than that of its bulb.The CN is a type of onion bulb that has been cultivated for over 2000 years in Campania region, especially in the areas of Pompeii-Nocera. It is characterized by several distinctive features. The harvested bulbs measure 2–4 cm, which classifies them as medium-small-sized onions. The bulb has a cylindrical shape and is flattened at the poles, with a slight thickening at the base of the leaves. The inner and outer layers of the bulb are completely white, and the flesh is succulent and sweet in taste. As a spring harvest onion (from March to June), it is primarily consumed fresh and does not have a high capacity for storage. The annual production is approximately 50,000 tons of fresh produce, resulting in a turnover exceeding 30 million euros. The CN has been granted the Protected Designation of Origin (PDO) status (Reg. CE n. 656/2008)^23^. Several studies have investigated the extraction of bioactive compounds from onion leaves, mainly using the conventional extraction technique^24–26^. These methods typically involve macerating the leaves in different solvents (e.g., ethanol, ethanol/water, or acetone) at varying times and temperatures to optimize the extraction process. This method takes a lot of time, energy, and solvent during processing^27–30^.

Recently, optimal conditions for ultrasound-assisted extraction (UAE) have been identified to obtain extracts from Welsh onion leaves. These extracts exhibit high polyphenol content and 2,2-Diphenyl-1-picrylhydrazyl (DPPH) scavenging activity^31^. UAE technique has gained popularity due to its ability to reduce solvent consumption, shorten extraction time, and improve extraction yields. UAE operates through the mechanical and cavitation effects generated by ultrasonic waves, which enhance the mass transfer of targeted compounds by breaking down the cell walls of the plant material^32,33^. However, ultrasonication system has disadvantages such as being expensive, occurring undesirable changes in molecules and requiring optimization. Another environmentally friendly extraction technique is microwave-assisted extraction (MAE), which involves the irradiation of samples soaked in a solvent. In contrast to conventional extraction methods, microwave irradiation can directly heat the reactants and solvent by passing through the walls of the reaction container. MAE is widely used in laboratories due to its numerous advantages. It helps reduce energy consumption and the amount of organic solvents required, leading to a decrease in waste generation. Its ability to efficiently extract bioactive compounds makes it a valuable tool in the field of natural product extraction and has gained considerable attention in scientific research and industrial applications^34,35^.

However, the complexity of mass transfer and the limited depth of microwave irradiation, influenced by factors including temperature and microwave frequency, present challenges for upscaling the MAE process. Therefore, achieving scale-up of MAE for industrial applications requires an in-depth analysis of how various parameters affect extraction kinetics. Consequently, it is crucial to develop models that can predict the optimum MAE conditions^36–38^.

In this current study, we assessed the phytochemical composition and antioxidant properties of CN leaves, aiming to unlock the potential value of this by-product (leaves) for nutraceutical, nutritional, and pharmacological uses. For these purposes, we developed and optimized an alternative method based on MAE for the recovery and isolation of bioactive compounds from CN leaves. A response surface methodology (RSM) through a Box–Behnken design (BBD) was applied, and model fit, regression equations, analysis of variance and 3D response curves were developed. Temperature (60–100 °C), time (5–25 min), extraction volume (6–12 mL) and ethanol concentration (40–80% v/v) were studied as the major parameters affecting the extraction efficiency and the antioxidant properties. A Box–Behnken design was adopted considering total phenolic content (TPC) and ferric reducing antioxidant power (FRAP) as responses. A Liquid Chromatography-High-Resolution Mass Spectrometry (LC-HRMS) platform was employed to elucidate the polyphenol profile of CN extract, which was obtained under the optimal extraction conditions determined by developed model. Additionally, in vitro evaluation of cell safety and the quenching of H_2_O_2_-induced intracellular reactive oxygen species (ROS) exerted by optimal CN extract were evaluated in a hepatocarcinoma cell line.

## Materials and methods

### Materials

Folin Ciocalteu’s reagent, gallic acid, sodium carbonate, 2,4,6-Trippyridyl-s-triazine (TPTZ), sodium acetate, acetic acid glacial, hydrochloric acid 37%, Iron(III) chloride hexahydrate, Iron(II) sulfate heptahydrate, 3-[4,5-dimethylthiazol-2,5-diphenyl-2H-tetrazolium bromide (MTT), 6-carboxy-2ʹ,7ʹ-dichlorodihydrofluorescein diacetate (DCFH-DA), hydrogen peroxide were obtained from Merck Life Science, Milan, Italy. All the solvents and additives LCMS grade were purchased from VWR Chemicals, Milan, Italy. CNs were kindly donated by consortium for the protection of “*Cipollotto* (spring onions) *Nocerino* DOP”.

### Methods

#### Sample preparation

Green onion stalks (leaves) were selected for the extraction of antioxidant compounds. The onion leaves used in this study are not from endangered species. The principles of experimental research and field studies on plants, including the collection of plant material, were conducted in accordance with relevant institutional, national, and international guidelines and legislation for plant material research. Subsequently, the collected samples were labelled, stored in a cooler and transported to the laboratory. The leaves were lyophilized for 24 h (Manifold Freeze Dryer MFDQ 2002, Laboquest, Westchester USA), using condenser temperature at − 80 °C and 1 Pa as vacuum pressure. After lyophilization, the dried leaves were milled into a powder and stored at − 20 °C until further analysis. Microwave-based extraction experiments were performed in a PreeKem-M3 digestion system equipped with an HP10 rotor (Preekem Scientific Instruments Co., Shanghai, China). The microwave frequency was set at 2450 MHz while the microwave power (watt) was automatically adjusted by the instrument’s program based on thermal conditions, time, and the number of vessels. Notably, it was determined that 100, 250 and, 500 W correspond to 60, 80 and, 100 °C, respectively. After MAE, CN extracts were centrifuged at 6000 rpm for 10 min at 4 °C (Mikro 220R centrifuge, Hettich, Germany) and the supernatants were frozen overnight at − 20 °C to facilitate the precipitation of interfering compounds. Finally, the extracts were freeze-dried, reconstituted with 1 mL of the corresponding extracting solvent, and subjected to spectrophotometric analysis.

The extraction yield for each run and for the optimal extract was calculated according to the following equation:

1 $$\text{Extraction yield (\%) } = \frac{\text{W}1}{\text{W}0}\times 100,$$

where W_1_ and W_0_ are the weights of the final dry extract and the initial sample, respectively.

Extraction yield data were reported in the supplementary information (Table S1).

### Optimization of extraction variables using Box–Behnken design and method validity testing

The relationship between four independent variables (A: temperature, 60–80–100 °C; B: time, 5–15–25 min; C: extraction volume, 6–9–12 mL; D: ethanol concentration, 40–60–80% v/v) and the dependent variables (responses) of total phenolic content (TPC, *Y1*) and reducing power (FRAP assay, *Y2*) was assessed using BBD-RSM modeling Each independent factor was associated with three distinct coded levels (− 1, 0, 1) (Table 1).

**Table 1:** Extraction variables selected for BBD optimization.

Independent variable	Symbols	Factor level			Dependent variable
		− 1	0	+ 1	
Temperature (°C)	A	60	80	100	*Y1*: TPC (mg GAE g^−1^ dw) *Y2*: FRAP (mmolFe(II)E g^−1^ dw)
Time (min)	B	5	15	25	
Extraction volume (mL)	C	6	9	12	
EtOH (%)	D	40	60	80	

A total of 29 experimental runs, comprising five central points, were generated. All experiments were performed randomly, and the range of the studied variables was selected according to preliminary tests and experimental limitations. All analyses were performed in triplicate (to calculate the reproducibility of the process) and the results were expressed as mean ± standard deviation (SD). RSM was performed using the Design Expert 11 software (Stat-Ease, Inc., Minneapolis, MN, USA) and the experimental data were subjected to regression analysis based on Eq. (2) to obtain quadratic polynomial empirical models:

2 $$Y = {\beta }_{0}+ \sum {\beta }_{i}{X}_{i}+ \sum {\beta }_{ii}{{X}_{i}}^{2}+ \sum \sum {\beta }_{ij}{X}_{i}{X}_{j},$$

where *Y* is the predicted response, *X*_*i*_ and *X*_*j*_ are independent variables, *β*_0_ is the intercept coefficient, *β*_*i*_ is the linear coefficient, *β*_*ii*_ is the quadratic coefficient, and *β*_*ij*_ is the interaction coefficient of *i* and *j* variables.

The response surface and contour plot approaches were used to visualize the correlation between responses and different levels of independent variables and interaction types between two independent variables.

A final confirmation experiment (n = 3) was performed using optimized independent extraction variables, and the experimental data were compared with predicted values for model validation.

The analysis of variance (ANOVA) method was employed, and the maximum R^2^ and adjusted R^2^ values were used to assess the accuracy of the estimated coefficients. A confidence level of 95% was adopted to determine the significance differences and *p-*values ≤ 0.05 considered to be significant.

### Total phenolic content analysis

The TPC of CN extract was determined using the Folin–Ciocalteau method as described by Way et al., with slight modifications^39^. Reagent A was prepared by combining 5 mL of 2 M Folin–Ciocalteu reagent to 45 mL of distilled water. For reagent B, 2.87 g of sodium carbonate was dissolved in distilled water in a 25 mL volumetric flask. For each sample, 2 μL of extract was added to 100 μL of reagent A in a microplate, mixed, and left for 5 min before adding 70 μL of reagent B and mixing. Then, the microplate was incubated for 1 h at 40 °C. The absorbance of the solution was then evaluated at 765 nm using a Multiskan SkyHigh Microplate Spectrophotometer (Thermo Fisher Scientific, Waltham, MA, USA). Gallic acid was selected as the standard. Stock solution (1 mg/mL) was prepared in MeOH, and the calibration curve was obtained in a concentration range of 10–200 mg/L, with five concentration levels (y = 991,17683x − 0.08039) and the linearity of the standard curve was 99.99%. The solution was measured in triplicate. The total phenolic content was calculated and expressed as milligrams of gallic acid equivalents per gram of dry weight (mg GAE g^−1^ dw).

### Ferric reducing antioxidant power assay

The FRAP method is based on the reduction of ferric ion (Fe^3+^) to ferrous ion (Fe^2+^). The assay was conducted with slight modifications to the conditions previously described by Noreen et al.^40^. FRAP reagent was prepared by mixing three solutions: A, 300 mM sodium acetate buffer, pH 3.6; solution B, 10 mM TPTZ solution in 40 mM HCl; and solution C, 20 mM ferric chloride (FeCl_3_) in a volumetric ratio of 10:1:1 v/v/v, respectively. The reagent was kept in darkness for 30 min to complete the reaction. Briefly, 5 μL of CN extracts were mixed with 145 μL of FRAP reagent. FeSO_4_ was used as analytical standard (0.1–5 mM; y = 2.71450x + 0.01491; R^2^ = 99.99%). All the samples were prepared in triplicate, shaked and incubated in dark for 30 min at 37 °C. Changes in the absorbance of the samples were measured against blank at 593 nm using a microplate reader. FRAP activity was calculated as millimoles of ferrous equivalent per gram of dry weight (mmol Fe(II)E g^−1^ dw).

### UHPLC-HRMS/MS conditions

UHPLC-HRMS/MS analysis was performed on a Thermo Ultimate RS 3000 coupled online to a Q-Exactive hybrid quadrupole Orbitrap mass spectrometer (Thermo Fisher Scientific, Bremen, Germany) equipped with a heated electrospray ionization probe (HESI II).

The separation was performed in reversed phase mode, with a Kinetex® 2.6 µm EVO C18 100 Å, 150 × 2.1 mm analytical column (Phenomenex, Bologna, Italy) thermostated at 40 °C. The mobile phases were H_2_O (A) and ACN (B) both acidified with 0.1 v/v % HCOOH delivered at a constant flow of 0.4 mL/min. The following gradient was employed: 0.01–25.00 min, 2–30% B; 25.01–35.00 min, 30–100% B; 35.01–37.00 min, isocratic to 100% B; 37.01–39.00 min, 2% B; then 5 min for column re-equilibration. 2 µL of CN extract were injected.

The ESI was operated both in negative and positive mode. The MS was calibrated by Thermo calmix Pierce™ calibration solutions in both polarities. Full MS (100–1500 m/z) and data-dependent MS/MS were performed at a resolution of 35,000 and 17,500 FWHM respectively, normalized collision energy (NCE) values of 15, 20, and 25 were used. Source parameters: Sheath gas pressure, 50 arbitrary units; auxiliary gas flow, 13 arbitrary units; spray voltage, + 3.5 kV, − 2.8 kV; capillary temperature, 310 °C; auxiliary gas heater temperature, 300 °C.

The identification of analyzed compounds was carried out by comparing their retention times and MS/MS data with those present in the literature. Data analysis and processing were performed using FreeStyle™ 1.8 SP2 and the commercial software Compound Discoverer v. 3.3.1.111 SP1 (Thermo Fisher Scientific, Bremen, Germany). The following online databases were also consulted: Phenol-Explorer (www.phenolexplorer.eu), PubChem (https://pubchem.ncbi.nlm.nih.gov), FooDB (https://foodb.ca/) and, ChemSpider (http://www.chemspider.com).

### Cell culture and drug treatment

The human hepatocarcinoma Hep G2 cell line was obtained from GMIST cell bank (Genova, Italy). Cells were grown in Eagle’s minimum essential medium, supplemented with 10% (v/v) fetal bovine serum (FBS), 1% (v/v) nonessential amino acid, 2 mM l-glutamine, 100 U/mL penicillin, and 100 mg/mL streptomycin.

Cells were routinely grown in culture dishes (Corning, Corning, NY) in a humidified atmosphere of 5% CO_2_/95% air at 37 °C and splitted every 2 days. The viability was monitored using phase contrast microscopy and trypan blue staining. In each experiment, cells were placed in a fresh medium and cultured in the presence of the optimal CN extract at different concentrations and times. Each treatment and analysis were performed in triplicate separate experiments. Cells were used at the 16–20th passage.

### Cell viability assay

Cell viability was established by measuring mitochondrial metabolic activity with MTT. Briefly, Hep G2 (30 × 10^3^ cells/well) were plated into 96-well plates, then CN extract (1.56–200 µg/mL) was added for 24 h. Afterward, MTT reagent (0.5 mg/mL) for 2 h was added. Then, 100 μL per well of 0.1 M isopropanol/HCl solution was added to dissolve formazan. The absorbance was measured at 570 nm, using a microplate reader (Multiskan Go, Thermo Scientific, Waltham, MA, USA). Cell viability was expressed as a percentage relative to the untreated cells cultured in medium with 0.1% DMSO and set to 100%, whereas 10% DMSO was used as positive control and set to 0% of viability. The EC_50_ values were calculated using GraphPad Prism 8.0 software by nonlinear regression of the dose–response inhibition.

#### Statistical analysis

Data are reported as mean ± SD of results from three independent experiments. Statistical analysis was performed using ANOVA test, and multiple comparisons were made with the Bonferroni’s test with GraphPad Prism 8.0 software (San Diego, CA, USA). Significance was assumed at *p* < 0.05.

### ROS detection

ROS levels were measured as previously described^41^. To test the effect of CN extract (50, 25 µg/mL) to ROS neutralization, Hep G2 cells were seeded (30 × 10^3^ cells/well) in black 96-well ViewPlate (PerkinElmer, USA) allowing to adhere for 24 h. Next, cells were incubated with both CN extract and H_2_O_2_ (800 μM) for 1 h. H_2_O_2_ alone (800 μM, 1 h) was used as positive control.

After treatments, the medium was removed, and the cells were washed with PBS. A staining solution containing 10 μM DCFH-DA in serum-free medium without phenol-red was added for 30 min at 37 °C in the dark. The fluorescence signals (excitation/emission 485 nm/535 nm) were read in end point mode using a PerkinElmer EnSpire multimode plate reader.

#### Statistical analysis

Data are reported as mean ± SD of results from three independent experiments. Statistical analysis was performed using ANOVA test, and multiple comparisons were made with the Bonferroni’s test with GraphPad Prism 8.0 software (San Diego, CA, USA). Significance was assumed at *p* < 0.05.

## Result and discussion

In this study, we examined the health benefits of onion wastes, specifically focusing on the leaves of a spring onion variety called “*Cipollotto Nocerino*” from the Campania Region. Our main objective was to investigate its potential as a source of antioxidant compounds.

MAE conditions for isolating antioxidant compounds were optimized using a BBD with a total of 29 runs. The study considered the influence of four independent variables: temperature, extraction time, ethanol concentration, and solvent volume. Table 2 shows the comprehensive experimental design, including the predicted and experimental values of TPC and FRAP.

**Table 2:** Experimental conditions for BBD the corresponding experimental and predicted values of TPC and FRAP.

Run	Factors				*Y1*: TPC (mg GAE g^−1^ dw)		*Y2*: FRAP (mmol Fe(II)E g^−1^ dw)	
	A	B	C	D	Predicted	Experimental	Predicted	Experimental
1	− 1	− 1	0	0	1.19	1.20	12.28	12.56
2	0	− 1	− 1	0	0.93	0.95	10.08	10.05
3	0	− 1	0	− 1	1.02	1.01	10.74	10.35
4	0	− 1	0	+ 1	1.15	1.19	11.38	11.48
5	0	− 1	+ 1	0	1.35	1.33	13.43	13.46
6	+ 1	− 1	0	0	1.19	1.15	11.49	11.50
7	− 1	0	− 1	0	0.98	0.95	9.76	9.92
8	− 1	0	0	− 1	1.11	1.14	11.62	11.50
9	− 1	0	0	+ 1	1.05	1.02	10.57	10.33
10	− 1	0	+ 1	0	1.29	1.29	13.82	13.89
11	0	0	− 1	− 1	0.86	0.84	8.58	8.69
12	0	0	− 1	+ 1	0.77	0.76	8.51	8.25
13	0	0	0	0	1.09	1.07	11.61	11.22
14	0	0	0	0	1.09	1.09	11.61	11.59
15	0	0	0	0	1.09	1.07	11.61	11.73
16	0	0	0	0	1.09	1.12	11.61	12.07
17	0	0	0	0	1.09	1.08	11.61	11.43
18	0	0	+ 1	− 1	0.98	0.98	12.11	12.24
19	0	0	+ 1	+ 1	1.28	1.28	12.59	12.35
20	+ 1	0	− 1	0	0.85	0.86	8.99	8.74
21	+ 1	0	0	− 1	0.83	0.86	9.34	9.90
22	+ 1	0	0	+ 1	1.10	1.06	10.80	11.24
23	+ 1	0	+ 1	0	1.18	1.22	12.54	12.19
24	− 1	+ 1	0	0	1.41	1.43	13.07	12.93
25	0	+ 1	− 1	0	1.13	1.15	10.18	10.47
26	0	+ 1	0	− 1	1.16	1.13	11.74	11.45
27	0	+ 1	0	+ 1	1.22	1.25	11.50	11.71
28	0	+ 1	+ 1	0	1.35	1.32	14.44	14.80
29	+ 1	+ 1	0	0	1.18	1.16	11.82	11.41

The TPC values range from 0.76 to 1.43 mg GAE g^−1^ dw, while the FRAP values range from 8.25 to 14.80 mmol Fe(II)E g^−1^ dw.

The experiments corresponding to five central points (runs: 13, 14, 15, 16, and 17) resulted in mean values of 1.09 ± 0.02 mg GAE g^−1^ dw (RSD = 1.91%), and 11.61 ± 0.32 mmol Fe(II)E g^−1^ dw (RSD = 2.76%) for TPC and FRAP, respectively, providing acceptable RSD values and an adequate agreement with the model.

According to the multiple regression analysis, the following quadratic polynomial empirical Eqs. (3) and (4), describing the relation between each response variable and the independent variables, were obtained; where A, B, C, and D correspond to temperature, extraction time, solvent volume, and ethanol, respectively.

3 $$ TPC = { 1}.0{87}0{1 } + - - 0.0{5998 } \times {\text{ A }} + \, 0.0{515779 } \times {\text{ B }} + \, 0.{159185 } \times {\text{ C }} + \, 0.0{5}0{3527 } \times {\text{ D }} + - - 0.0{561667 } \times {\text{ AB}} + \, 0.0{82}0{612 } \times {\text{ AD }} + - - 0.0{53}000{4 } \times {\text{ BC}} + \, 0.0{962268 } \times {\text{ CD}} + \, 0.{135}0{82 } \times {\text{ B}}^{{2}} + - - 0.0{3266}0{7 } \times {\text{ C}}^{{2}} + - - 0.0{841261 } \times {\text{ D}}^{{2}} , $$

4 $$ FRAP = { 11}.{6}0{53 } + - - 0.{511671 } \times {\text{ A }} + \, 0.{28}0{19 } \times {\text{ B }} + { 1}.{9}0{122 } \times {\text{ C}} + \, 0.{629}0{48 } \times {\text{ AD }} + \, + \, 0.{658719 } \times {\text{ B}}^{{2}} + - - 0.{924372 } \times {\text{ D}}^{{2}} . $$

### Influence of operational parameters on total phenolic content and ferric reducing antioxidant power

Table 3 shows the ANOVA results for RSM models used to analyse the TPC and FRAP responses.

**Table 3:** Analysis of variance for the independent variables *Y1* (TPC) and *Y2* (FRAP) studied in the extraction of CN by the experimental treatments.

Source	TPC (mg GAE g^−1^ dw)					FRAP (mmol Fe(II)E g^−1^ dw)				
	Sum of squares	Degree of freedom	Mean square	F-value	*p*-value	Sum of squares	Degree of freedom	Mean square	F-value	*p*-value
Model	0.7114	14	0.0508	43.49	< 0.0001*	60.23	14	4.30	28.33	< 0.0001*
A-Temp.	0.0432	1	0.0432	36.95	< 0.0001*	3.14	1	3.14	20.69	0.0005*
B-Time	0.0319	1	0.0319	27.32	0.0001*	0.9421	1	0.9421	6.20	0.0259*
C-Extr. Vol.	0.3041	1	0.3041	260.23	< 0.0001*	43.38	1	43.38	285.67	< 0.0001*
D-EtOH	0.0304	1	0.0304	26.04	0.0002*	0.1251	1	0.1251	0.8240	0.3794
AB	0.0126	1	0.0126	10.80	0.0054*	0.0525	1	0.0525	0.3461	0.5657
AC	0.0001	1	0.0001	0.0841	0.7761	0.0665	1	0.0665	0.4380	0.5188
AD	0.0269	1	0.0269	23.05	0.0003*	1.58	1	1.58	10.42	0.0061*
BC	0.0112	1	0.0112	9.62	0.0078*	0.2106	1	0.2106	1.39	0.2586
BD	0.0010	1	0.0010	0.8579	0.3700	0.1893	1	0.1893	1.25	0.2830
CD	0.0370	1	0.0370	31.70	< 0.0001*	0.0749	1	0.0749	0.4934	0.4939
A^2^	0.0025	1	0.0025	2.18	0.1624	0.0611	1	0.0611	0.4021	0.5362
B^2^	0.1184	1	0.1184	101.29	< 0.0001*	2.81	1	2.81	18.54	0.0007*
C^2^	0.0069	1	0.0069	5.92	0.0290*	0.3434	1	0.3434	2.26	0.1548
D^2^	0.0459	1	0.0459	39.29	< 0.0001*	5.54	1	5.54	36.50	< 0.0001*
Residual	0.0164	14	0.0012			2.13	14	0.1518		
Lack of fit	0.0146	10	0.0015	3.37	0.1267	1.72	10	0.1715	1.67	0.3279
Pure error	0.0017	4	0.0004			0.4107	4	0.1027		
R^2^	0.9775					0.9659				
Adjusted R^2^	0.9550					0.9318				
C.V. %	3.10					3.43				

*Significant at *p* < 0.05.

The F-values of 43.49 (TPC) and 28.33 (FRAP) and *p-*values less than 0.05 indicate model terms are significant. The quadratic coefficients B^2^, C^2^ and D^2^ as well as the interaction coefficient AB, AD, BC, CD were significant in the model developed for total phenolic content (*p* < 0.05) while that factors A, B, C, AD, B^2^ and D^2^ had significant effects (*p* < 0.05) on the reducing power.

In addition, the high R^2^ (0.98, and 0.97 for TPC, and FRAP, respectively) and Adj-R^2^ values (TPC: 0.96; FRAP: 0.93), the coefficient of variation CV (TPC: 3.10; FRAP: 3.43) and the non-significant values for lack of fit (*p* > 0.05, TPC: 0.13; FRAP: 0.33) confirmed that the mathematical model of equations was able to predict the total phenolic content and antioxidant properties according to the various combination of variables values.

**Figure 1:**
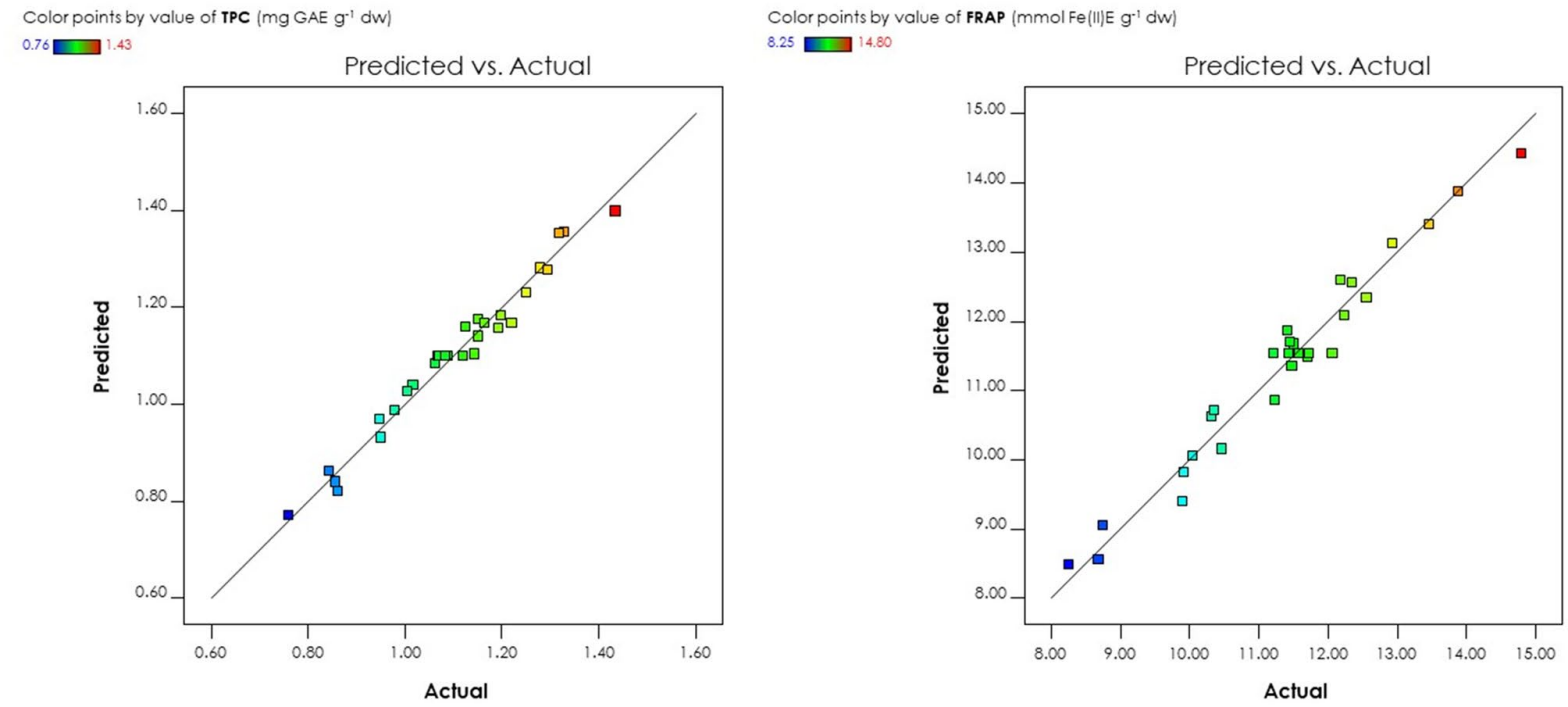
Diagnostic plot obtained by the BBD of predicted values versus actual values for TPC (left) and FRAP (right).

Additionally, the accuracy of the regression model was assessed by evaluating the Diagnostic plot of predicted vs. actual values. The comparison between the predicted and actual response, as showed in Fig. 1, confirms that the experimental values align closely with the predicted values, indicating a good fit without any significant deviations.

**Figure 2:**
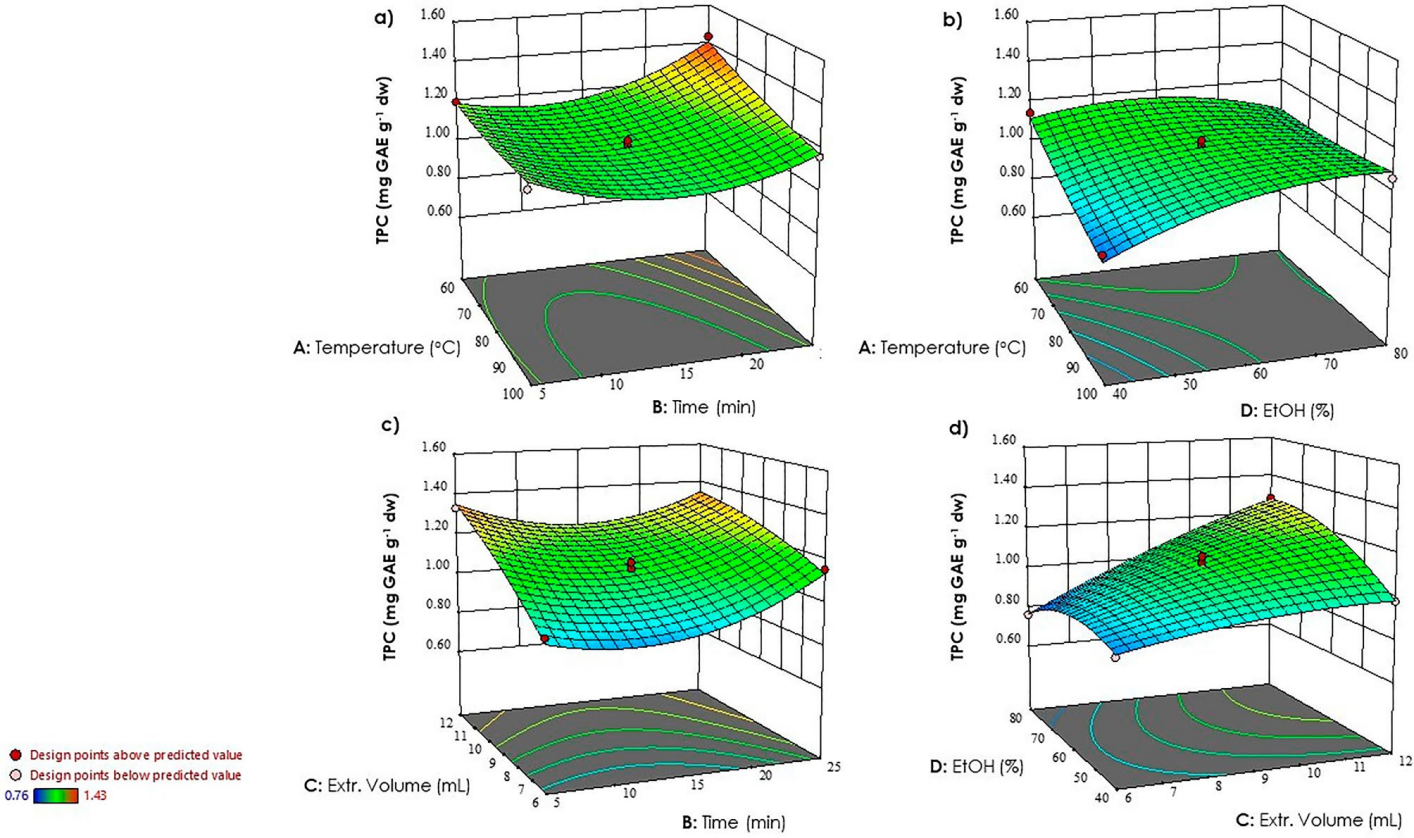
Three-dimensional surface plots were utilized to illustrate the interactions among various process variables on TPC: (**a**) temperature *vs* time; (**b**) temperature *vs* EtOH concentration; (**c**) extraction volume *vs* time; (**d**) EtOH concentration *vs* extraction volume.

**Figure 3:**
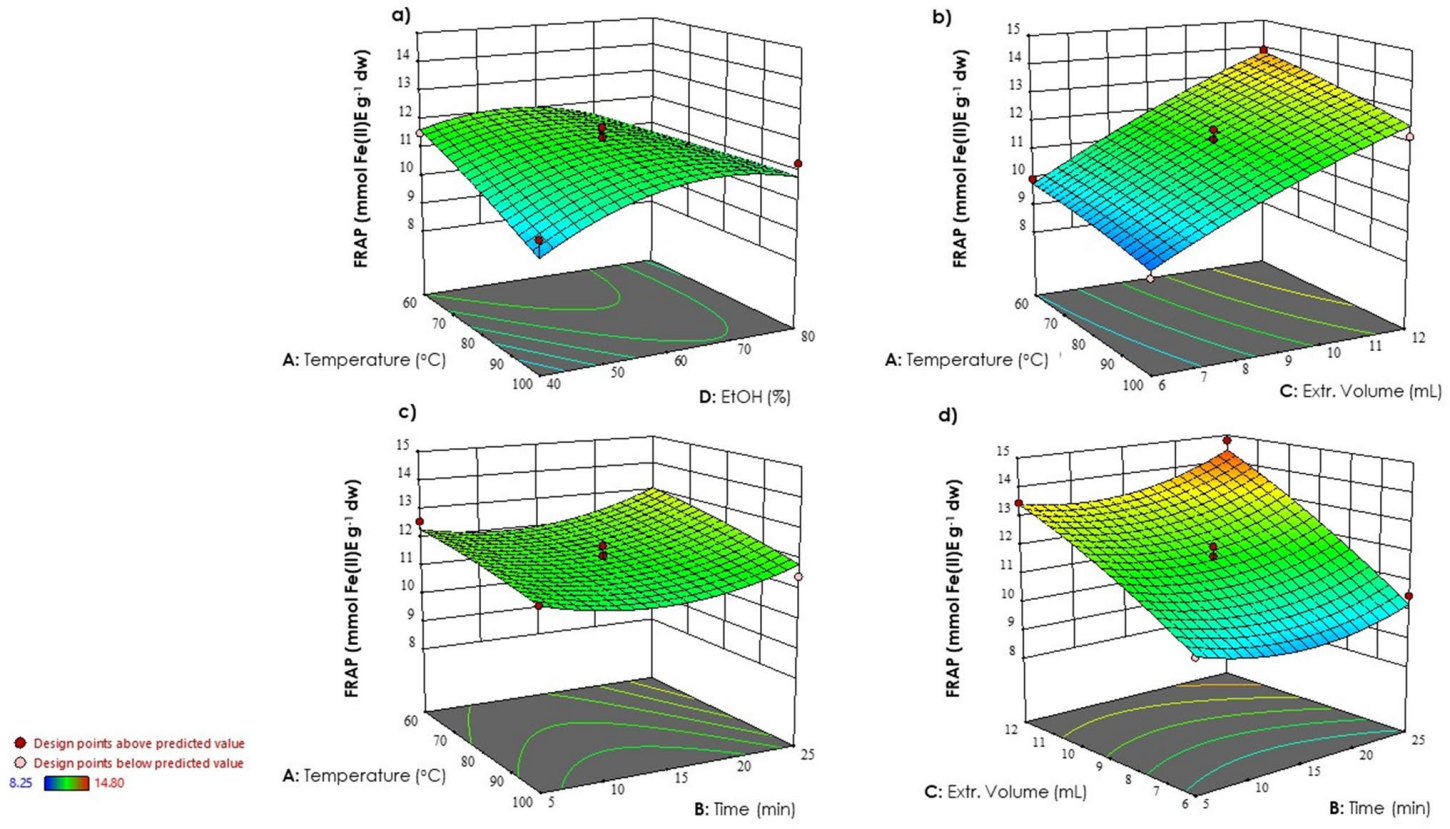
Response surface plots showing significant interactions between independent variables on FRAP: (**a**) temperature *vs* EtOH concentration; (**b**) temperature *vs* extraction volume; (**c**) temperature *vs* time; (**d**) extraction volume *vs* time.

The response surface plots showed the impact of different process variables on TPC (Fig. 2) and FRAP (Fig. 3) values.

A significant positive interaction was observed between time and extraction volume (Figs. 2c, 3d), enhancing TPC and antioxidant activity. It is well-known that an increase in time and extraction volume can enhance the solubility of polyphenolic compounds from vegetable matrices, facilitating their diffusion into the extraction solvent^42^.

These findings are in line with previous studies, wherein it was demonstrated that high amount of solvent increases its penetration through the cell wall by causing swelling in the cell wall and membrane^43^. This phenomenon enhances the permeability of solvent molecules into the cell, resulting in a stronger interaction between extraction solvent and phytochemicals^44^. It has been reported that the polar nature of phenolic compounds is influenced by an increased polarity index of the solvent, which is attributed to the increased solvent volume, consequently this leads to an increase in the extraction of these bioactive compounds^45^.

In MAE, treatment time plays a pivotal role in influencing the extraction of bioactive compounds from the plant material^46,47^. When samples are exposed to microwave radiation for a longer time the greater disruption of cell walls occurs, facilitating greater mass transfer from the interior of the sample to the solvent, resulting in an efficient increase in TPC^48–50^. Excessive exposure to microwave irradiation can lead to overheating of the plant material. Optimization is crucial to determine the optimal duration of microwave treatment^51^.

The negative impact of ethanol concentration was also observed in the response surfaces, as depicted in Figs. 2d and 3a. The response values were observed to be higher when the ethanol concentration was closer to the lower values. According to Yang et al.^52^, at lower concentrations, ethanol can penetrate the plant cells more easily and facilitate the extraction of polyphenolic compounds^53^. On the other hand, higher concentrations of ethanol may lead to protein denaturation and dehydration of the plant cells, hindering the extraction process and resulting in lower yields^54,55^.

Higher temperatures were associated with lower response values for both total phenolic content (Fig. 2a,b) and antioxidant activity (Fig. 3a,b). This is due to thermal degradation of sensitive compounds at elevated temperatures. Increased kinetic energy at higher temperatures can break down or alter the structure of target compounds, reducing extraction efficiency^56^.

### Simultaneous multi-response optimization

The optimal conditions for MAE were determined using a numerical optimization approach. Numerical optimization ramps were employed to determine the optimum values for temperature, extraction time, extraction volume, and ethanol concentration, with the objective of maximizing the response variables. The optimal MAE conditions were determined using desirability as a criterion. Based on RSM, the optimum conditions were found to be a temperature of 60 °C for 22 min, using 11 mL of 51% (v/v) ethanol, with a desirability score of 0.924 (Table 4). To validate the predicted response variables, experimental assays were conducted in triplicate under these optimal conditions. The experimental results obtained for TPC, and antioxidant activity (FRAP) were in close agreement with the values predicted by the polynomial quadratic models, indicating the reliability and effectiveness of the optimized MAE-BBD-RSM method.

**Table 4:** Experimental values and predicted values of response variables at optimum extraction conditions.

	Independent variable			
	Temperature (°C)	Time (min)	Extr. volume (mL)	EtOH (%v/v)
Optimal conditions	60.000	22.000	11.000	50.990

Responses	TPC (mg GAE g^−1^ dw)			FRAP (mmol Fe(II)E g^−1^ dw)
Predicted values	1.334			14.110
Expertimental results*	1.351 ± 0.07			14.016 ± 0.24
*p*-value^#^	0.7149			0.5675

*Mean values ± standard deviation (*n* = 3).

^#^Significant at *p* < 0.05.

This approach allows for the reduction in the number of experiments required for polyphenol extraction with antioxidant properties without compromising the validity of the results.

### Chemical profile of CN optimal extract

After identifying the optimal conditions for MAE, the next step was to characterize the extract obtained under optimized parameters (60 °C; 22 min; 11 mL; 51% v/v EtOH). This characterization aimed to assess the composition and properties of the extract, providing valuable information about its chemical profile and potential bioactive components. The liquid chromatography–mass spectrometry method was optimized, operating under both, negative and positive modes, to find the best fragmentation pattern for each compound (Fig. S1). LC/MS–MS platform allowed us to tentatively identify a total of 63 compounds, primarily belonging to the class of flavonoids, saponins, fatty acids, and lipids.

The complete list of the compounds tentatively identified in optimal CN extract is reported in Table 5.

**Table 5:** Complete list of tentatively compounds identified in CN extract.

Peak	Compound	Rt (min)	[M − H]^−^	[M + H]^+^	MS/MS	Chemical formula	Error (ppm)	Class	References
1.	Quercetin 7,4′-dihexoside	7.69	625.1412	–	463.0884; 300.0274; 301.0352	C_27_H_30_O_17_	1.80	Flavonoids	^57,58^
2.	Herniarin	8.39	–	177.0544	145.0282	C_10_H_8_O_3_	− 1.52	Coumarin	^59^
3.	Kaempferol 3,7-*O*-dihexoside	8.45	609.1461	–	285.0404;	C_27_H_30_O_16_	1.62	Flavonoids	^60^
4.	Kaempferol 3,7-*O*-dihexoside *(isomer I)*	12.03	609.1458	–	285.0326; 178.9979; 151.0025	C_27_H_30_O_16_	1.82	Flavonoids	^60^
5.	Quercetin 3,4′-dihexoside	12.61	–	627.1539	303.0494; 465.1018	C_27_H_30_O_17_	− 2.43	Flavonoids	^60^
6.	Cyanidin 3-laminaribioside	12.76	–	611.1595	449.1070; 287.0546	C_27_H_30_O_16_	− 2.02	Flavonoids	^57^
7.	Quercetin 3,4′-dihexoside *(isomer I)*	13.22	625.1411	–	300.0274; 301.0353	C_27_H_30_O_17_	1.70	Flavonoids	^57^
8.	Quercetin 3-*O*-hexoside	13.39	463.0881	–	301.0353; 178.9982; 151.0023	C_21_H_20_O_12_	2.30	Flavonoids	^60,61^
9.	Kaempferol	13.95	–	287.0546	165.0176; 153.0178	C_15_H_10_O_6_	− 1.56	Flavonoids	^60^
10.	Phenethyl rutinoside	14.03	429.1765	–	267.1237; 223.1335	C_20_H_30_O_10_	0.74	Glycoside	^61^
11.	Quercetin	14.73	–	303.0495	229.0468; 253.0465	C_15_H_10_O_7_	− 2.12	Flavonoids	^60^
12.	Kaempferol 3,7-*O*-dihexoside *(isomer II)*	15.51	609.1463	–	484.1300; 285.0404; 151.0025	C_27_H_30_O_16_	2.22	Flavonoids	^60^
13.	Kaempferol 3-*O*-hexoside	15.72	447.0936	–	327.0512; 284.0328	C_21_H_20_O_11_	2.88	Flavonoids	^60^
14.	Quercetin 3-*O*-hexoside* (isomer I)*	15.79	463.0887	–	301.0352; 178.9977; 151.0023	C_21_H_20_O_12_	1.83	Flavonoids	^60–61^
15.	Kaempferol 3-*O*-hexoside *(isomer I)*	16.17	447.0934	–	327.0522; 285.0404	C_21_H_20_O_11_	2.54	Flavonoids	^60^
16.	Isorhamnetin-*O*-hexoside	16.38	477.1035	–	357.0601; 314.0431; 178.9971; 151.0025	C_22_H_22_O_12_	1.60	Flavonoids	^62,63^
17.	Kaempferol *(isomer I)*	16.47	–	287.0547	153.0182	C_15_H_10_O_6_	− 1.67	Flavonoids	^60^
18.	Quercetin-3-*O*-feruloyl-sophoroside-7-*O*-d-glucoside	16.55	963.2421	–	787.1927; 301.0350; 178.9975	C_43_H_48_O_25_	2.11	Flavonoids	^63^
19.	Petunidin 3-hexoside	17.02	–	479.1174	317.0649; 303.0494	C_22_H_23_O_12_^+^	− 1.90	Flavonoids	^64^
20.	Quercetin-3-*O*-feruloyl-sophoroside-7-*O*-d-glucoside *(isomer I)*	17.17	963.2423	–	787.1950; 301.0354; 178.9978	C_43_H_48_O_25_	2.43	Flavonoids	^63^
21.	Kaempferol-3-*O*-coumaroyldiglucoside-7-O-glucoside	17.25	917.2365	–	771.1991; 591.1382; 284.0326	C_42_H_46_O_23_	2.07	Flavonoids	^63^
22.	Isorhamnetin-*O*-hexoside *(isomer I)*	17.44	477.1043	–	357.0813; 314.0433; 153.0186	C_22_H_22_O_12_	2.05	Flavonoids	^62,63^
23.	Kaempferol-3-*O*-feruloyldiglucoside-7-*O*-glucoside	17.62	947.2475	–	771.2000; 489.1023; 284.0327	C_43_H_48_O_24_	2.26	Flavonoids	^63^
24.	Kaempferol-3-*O*-coumaroyldiglucoside-7-*O*-glucoside *(isomer I)*	17.73	917.2368	–	771.1994; 591.1359; 284.0328	C_42_H_46_O_23_	2.27	Flavonoids	^63^
25.	Kaempferol-3-*O*-feruloyldiglucoside-7-*O*-glucoside *(isomer I)*	18.03	947.2476	–	771.1990; 284.0327	C_43_H_48_O_24_	2.19	Flavonoids	^63^
26.	Saponin 3-IV4-1 (447 + dHex + 2 Hex + FA)	19.42	963.4808	–	917.4761; 771.4178; 609.3665	C_46_H_75_O_21_	1.35	Saponins	^65^
27.	Neohecogenin-3- Oβ-Dglucopyranosyl (1 → 2)-β-d-glucopyranosyl (1 → 4)-β-d-galactopy-ranoside	19.54	–	901.4769	269.1896; 287.2003; 413.3043; 595.3106	C_45_H_72_O_18_	− 1.78	Steroidal glycosides	^66^
28.	Saponin 3-IV4-1 *(isomer I)* (447 + dHex + 2 Hex + FA)	19.71	963.4818	–	917.4764; 771.4146; 609.3652	C_46_H_75_O_21_	2.30	Saponins	^65^
29.	Neohecogenin-3-Oβ-Dglucopyranosyl (1 → 2)-β-d-glucopyranosyl (1 → 4)-β-d-galactopy-ranoside *(isomer I)*	19.79	–	901.4769	269.1896; 287.2003; 413.3046; 595.3112	C_45_H_72_O_18_	− 1.85	Steroidal glycosides	^66^
30.	7-Hydroxy-2’,4’,5-trimethoxyflavanone	19.92	329.1031	–	135.0440; 193.0498	C_18_H_18_O_6_	3.14	Flavonoids	^67^
31.	Saponin 3-IV4-2 (447 + Pen + dHex + Hex + FA)	20.14	933.4701	–	887.4649; 741.4050; 609.3657; 447.3091	C_45_H_73_O_20_	1.20	Saponins	^65^
32.	Pennogenin-3-*O*-α-L-arabinofuranosyl(1 → 4)[α-l-rhamnopyranosyl(1 → 2)]-β-d-glucopyranoside	20.25	–	871.4655	269.1895; 287.2003; 709.4147; 413.3044	C_44_H_70_O_17_	− 2.11	Steroidal glycosides	^66^
33.	Quercetin-3-*O*-feruloyl-sophoroside	22.15	801.1894	–	625.1414; 300.0276; 445.0790	C_37_H_38_O_20_	1.66	Flavonoids	^63^
34.	Kaempferol *(isomer II)*	23.31	285.0406	–	–	C_15_H_10_O_6_	3.44	Flavonoids	^62^
35.	Oxo-dihydroxy-octadecenoic acid (oxoDiHODE)	24.43	327.2177	–	309.2067; 229.1442; 211.1334; 183.1386	C_18_H_32_O_5_	3.12	Fatty acids	^68^
36.	9,12,13-Trihydroxy octadeca-7-enoic acid (TriHODE)	26.46	329.2233	–	311.2212; 229.1442; 211.1333	C_18_H_34_O_5_	2.93	Fatty acids	^62,68^
37.	9,12,13-Trihydroxy octadeca-7-enoic acid (TriHODE) *(isomer II)*	26.59	329.2234	–	311.2224; 293.2100; 229.1442; 211.1333	C_18_H_34_O_5_	3.02	Fatty acids	^62,68^
38.	Saponin 2-III4 (445 + dHex + Pen + FA)	26.85	769.4023	–	723.3962; 577.3372; 445.2929	C_39_H_61_O_15_	4.30	Saponins	^65^
39.	9,12,13-Trihydroxy octadeca-7-enoic acid (TriHODE) *(isomer III)*	27.06	329.2235	–	311.2211; 293.2118; 229.1448; 221.1335	C_18_H_34_O_5_	3.30	Fatty acids	^62,68^
40.	Palmitoylglycine	27.33	–	314.2688	240.2318; 296.2585	C_18_H_35_O_3_N	− 0.84	Fatty acids	^61^
41.	Palmitoylglycine *(isomer I)*	27.70	–	314.2686	296.2583; 72.0450	C_18_H_35_O_3_N	− 1.13	Fatty acids	^61^
42.	2′-Hydroxy-4,4′,6′-trimethoxychalcone	27.77	313.1083	–	193.0499	C_18_H_18_O_5_	4.11	Flavonoids	^67^
43.	5,6,7,4′-Tetramethoxyflavanone	28.01	343.1189	–	193.0499	C_19_H_20_O_6_	2.94	Flavonoids	^69^
44.	Dehydrophytosphingosine	28.15	–	316.2839	298.2740; 280.2636	C_18_H_37_O_3_N	− 1.39	Sphingolipids	^61^
45.	Palmitoylglycine *(isomer II)*	28.53	–	314.2686	296.2584; 72.0449	C_18_H_35_O_3_N	− 1.03	Fatty acids	^61^
46.	Dehydrophytosphingosine *(isomer I)*	28.60	–	316.2844	298.2738; 280.2632	C_18_H_37_O_3_N	− 1.59	Sphingolipids	^61^
47.	Phytosphingosine	29.03	–	318.2998	60.0450; 300.2896; 282.2790	C_18_H_39_O_3_N	− 1.18	Sphingolipids	^69^
48.	Tigogenin	30.23	415.3178	–	311.3078; 371.3276	C_27_H_44_O_3_	− 6.52	Sapogenin	^70^
49.	Saponin 2-III3 (429 + dHex + Pen + FA)	30.39	753.4067	–	707.4014; 561.3437; 429.3004	C_39_H_61_O_14_	0.80	Saponins	^65^
50.	Hydroxyoctadecatrienoic acid (HOTrE)	31.15	293.2124	–	275.2017; 195.1385	C_18_H_30_O_3_	3.85	Fatty acids	^67,71^
51.	LysoPC(16:0)	31.64	–	496.3390	184.0732; 104.1072; 86.0968	C_24_H_50_O_7_NP	− 1.26	Glycerophospholipids	^72,73^
52.	13-Hydroxyoctadecadienoic acid (13-HODE)	31.65	295.2278	–	277.2173; 195.1383	C_18_H_32_O_3_	3.12	Fatty acids	^74^
53.	13-Hydroxyoctadecadienoic acid *(isomer I)*	31.84	295.2276	–	277.2172; 195.1376	C_18_H_32_O_3_	2.91	Fatty acids	^74^
54.	α-Linolenoyl ethanolamide	32.14	–	322.2743	62.0607; 305.2481	C_20_H_34_O_2_N	− 0.97	Fatty amide	^69^
55.	Linoleoyl ethanolamide	32.86	–	324.2894	62.0607; 307.2631; 263.2371; 245.2260	C_20_H_37_O_2_N	− 0.85	Fatty amide	^69^
56.	Hydroxy-hexadecanoic acid	33.42	271.2279	–	225.2219	C_16_H_32_O_3_	3.73	Fatty acids	^62,67^
57.	3-Dehydrosphinganine (C20)	33.58	–	326.3048	62.0606; 309.2787	C_20_H_39_O_2_N	− 1.30	Sphingolipids	^67^
58.	Hexadecanamide	33.74	–	256.2631	–	C_16_H_33_ON	− 1.80	Fatty amide	^67^
59.	Sphingosine	34.02	–	282.2790 [M + H-H_2_O]	265.2523	C_18_H_37_O_2_N	− 0.75	Sphingolipids	^67,69^
60.	Pheophorbide a	34.63	–	593.2750	533.2538	C_35_H_36_O_5_N_4_	− 1.49	Chlorophylls	^75^
61.	Octadecanamide	34.91	–	284.2944		C_18_H_37_ON	− 0.08	Fatty amide	^67^
62.	1,3-Dilinolenoylglycerol (DG(18:3n6/0:0/18:3n6))	35.23	–	613.4813	595.4719	C_39_H_64_O_5_	− 0.97	Glycerolipids	^67,69^
63.	1,3-Dilinolenoylglycerol (DG(18:3n6/0:0/18:3n6)) *(isomer I)*	35.65	–	613.4821	595.4711	C_39_H_64_O_5_	− 1.07	Glycerolipids	^67,69^

*Hex* hexosyl, *dHex* deoxyhexosyl, *Pen* pentosyl, *FA* formic acid.

Among the identified flavonoids (28), CN extract was found to be rich in flavonol glycosides with kaempferol and quercetin as the main aglycones.

According to the negative fragmentation pattern, peaks **3**,** 4**, and **12** were tentatively identified as isomers of Kaempferol 3,7-*O*-dihexoside, with a molecular ion [M − H]^−^ at *m/z* 609. In MS2, these compounds exhibited a disaccharide moiety, and the loss of 324 Da resulted in the aglycone deprotonated ion at *m/z* 285. Peaks **13** and **15** were tentatively identified as isomer of Kaempferol attached to a single sugar moiety. The [M − H]^−^ ion at *m/z* 447 corresponded to the molecular formula C_21_H_20_O_11_, and it produced fragment ions at *m/z* 285 [M − H-162]^−^. Compounds **17** and **34** were tentatively identified as kaempferol in positive and negative ionization mode, respectively.

The chromatogram of CN analyzed in positive ionization mode showed a **peak 5** with [M + H]^+^ ion at *m/z* 627. Fragment ions at *m/z* 465 and at *m/z* 303 were observed, corresponding to the loss of a sugar moiety [M + H–162]^+^ and to the aglycone portion, respectively. This peak was identified as Quercetin 3,4ʹ-didihexoside. Peaks **8** and **14** showed a molecular ion at *m/z* 463 [M − H]^−^, they have been identified as isomers of Quercetin 3-*O*-hexoside. The deprotonated molecular ion further generated an ion at *m/z* 301 through the relative loss of sugar moiety (− 162 Da)^57,60,61^.

Several fatty acids were identified in CN extract. Peak **35** showed at *m/z* 327 [M − H]^−^ a fragmentation ions at *m/z* 309 and *m/z* 229 produced by loss of water molecule and end-group HO-CH=CH(CH_2_)_3_CH_3_, respectively. This compound was tentatively identified as oxo-dihydroxy-octadecenoic acid (oxoDiHODE). A similar fragmentation pattern was also observed for 9,12,13-trihydroxy octadeca-7-enoic acid (TriHODE). Peaks **36**, **37** and **39** which were detected at different retention times in the chromatogram, all exhibited ions at *m/z* 329 [M − H]ˉ. The MS/MS fragmentation pattern showed ions at *m/z* 311 [M − H–H_2_O]ˉ, 293 [M − H–H_2_O-H_2_O]ˉ and 229 [M – H–100]ˉ corresponding to the loss of water and end-group HO-CH=CH(CH_2_)_3_CH_3_, respectively^62,68^.

Chromatographic peaks **52** and **53** exhibited the precursor ion at *m/z* 295, but the loss of a water molecule [M − H–18]^−^ and the relative cleavage of the C=C bond adjacent to the hydroxyl group gave fragments at *m/z* 277 and *m/z* 195, leading to its tentative identification as 13-hydroxyoctadecadienoic acid (13-HODE)^74^.

LC-HRMS/MS in negative ionization mode, enabled the detection of five putative saponins in CN extracts. Saponins were observed as deprotonated formic acid (FA) adducts and the fragmentation pattern generally corresponding to the neutral loss of FA (46 Da) and/or glycosyl moieties, i.e. hexosyl (Hex), deoxyhexosyl (dHex), pentosyl (Pen) (Table S1). In addition, it was possible to tentatively identify saponins aglycon ions (sapogenin) by analysing diagnostic fragments associated with the sequential loss of the FA and glycosyl groups.

Peaks **26** and **28** exhibited an [M − H]^−^ ion at *m/z* 963 (C_46_H_75_O_21_) with MS/MS fragments at *m/z* 917, 771 and, 609 corresponding to the successive loss of formic acid (46 Da), deoxyhexosyl (C_6_H_10_O_4_) and hexosyl groups (C_6_H_10_O_5_, *m/z* 162), respectively. Thus, after the loss of dHex + Hex + FA, the unresolved portion was tentatively identified as sapogenin IV4 (C_27_H_43_O_5_, *m/z* 447), along with an additional hexosyl moiety. Based on these findings, these peaks were identified as Saponin 3-IV4-1.

Saponin 2-III3 (compound **49**) showed a precursor ion at *m/z* 753 (C_39_H_61_O_14_) and generated MS/MS base fragment ions at *m/z* 707 and *m/z* 561 through the loss of formic acid (46 Da) and deoxyhexosyl (146 Da), respectively. Furthermore, a sequential cleavage of a pentosyl moiety (132 Da) resulted in a putative identification as sapogenin III3 (C_27_H_41_O_4_, *m/z* 429)^65^.

Phytosphingosine (peak **47**) and Dehydrophytosphingosine (peak **44, 46**) were detected in the samples analyzed using positive ionization mode. Peak **47** showed a precursor ion at *m/z* 318 [M + H]^+^. The most common fragments associated with this molecule were observed after the loss of a water molecule, resulting in the fragment ion at *m/z* 300 [M + H–H_2_O]^+^. Subsequently, the loss of another water molecule, led to the formation of the *m/z* 282 fragment [M + H–H_2_O–H_2_O]^+^. A similar fragmentation pattern was observed for Dehydrophytosphingosine (*m/z* 316, [M + H]^+^), where two consecutive losses of water molecules were observed, resulting in the formation of two fragments at *m/z* 298 [M + H–H_2_O]^+^ and *m/z* 280 [M + H–H_2_O–H_2_O]^+^.^61^.

### Optimal CN extract protects HepG2 cells from oxidative stress induced by hydrogen peroxide

The antioxidant properties of CN extracts were assessed using two different cell-free assay, DPPH (Table S1) and FRAP tests. DPPH assay is the most used antioxidant assay for plant extracts. In this assay, a molecule or antioxidant with weak A-H bonding will react with a stable free radical DPPH· causing its discoloration^76^.

FRAP test is a chemical method used to assess the antioxidant activity of a sample in vitro. It is based on the sample’s ability to reduce a Fe^3+^ complex of tripyridyltriazine (Fe(TPTZ)^3+^) to Fe(TPTZ)^2+^ which is intensely in blue color at low pH^77^. Although these antioxidant assay are based upon well-known chemical reactions, this probably do not reflect the cellular physiological conditions^78^. Indeed, an antioxidant is not only a substance able to prevent another substrate from oxidation, but a molecule that protects the whole biological system from damages coming from oxidizing stressors^79,80^.

For these reasons, we evaluated the antioxidant activity of CN in hepatocarcinoma cell line Hep G2 treated with hydrogen peroxide. Firstly, in vitro cytotoxicity of CN extract by measuring the cell viability of Hep G2 using MTT assay was evaluated. Hep G2 is a popular hepatic cell line used in a broad range of biochemical applications, including cytotoxic studies since it is widely employed as in vitro model to study liver functions^81^. Hep G2 cells were incubated with CN extract for 24 h at different concentrations (1.56–200 µg/mL) followed by morphology evaluation and determination of cell mortality.

**Figure 4:**
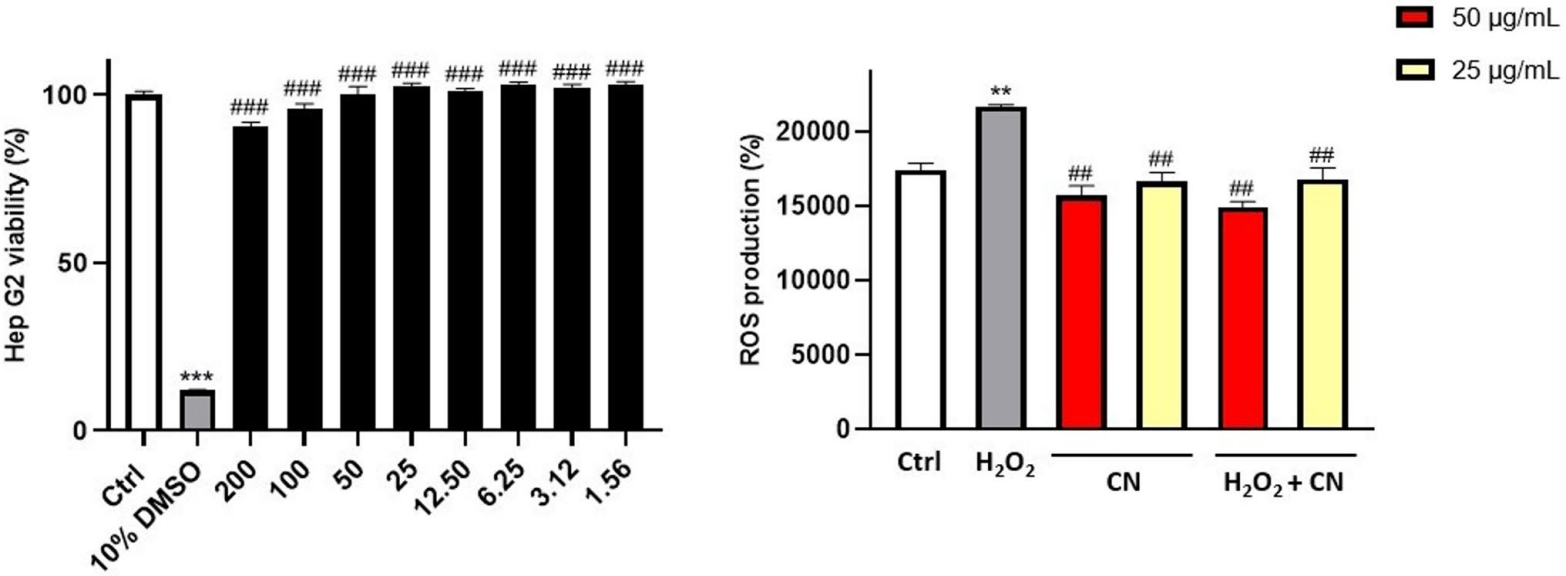
(Left) Cell safety evaluation of CN extract. Cell viability was performed using MTT assay. 10% DMSO was used as positive control. (Right) Measurement of intracellular ROS detected with DCFH-DA. H_2_O_2_ (800 μM, 1 h) was used as positive control. Data are showed as the mean ± SD of three different experiments performed in triplicate. **p < 0.01 vs. Ctrl; ***p < 0.001 vs. Ctrl; ^##^*p* < 0.01 vs. H_2_O_2_; ^###^*p* < 0.01 vs. 10% DMSO.

As shown in Fig. 4, the viability of cells treated without CN extract was defined as 100% (control group). 10% DMSO was used as positive control of mortality showing 12.54% of viability. The relative cell viabilities were always very high (viability > 90.64%) showing no cytotoxicity of CN extract compared to positive control.

Once the cell safety of optimal CN extract had been demonstrated, we proceeded to evaluate its ability to reduce intracellular release of ROS, induced by hydrogen peroxide. Our data highlighted that CN optimal extract (50–25 µg/mL) significantly inhibited ROS release in a concentration dependent manner in Hep G2 cells treated with H_2_O_2_.

Based on the obtained results, it can be concluded that spring onion leaves, which are considered an agricultural by-product, are a valuable source of antioxidant compounds. This finding suggests that they hold potential as functional ingredients for the production of new value-added products, such as functional foods and dietary supplements.

## Conclusion

In the present study, we investigated, for first time, the nutraceutical potential of green onion stalks, a by-product of *Cipollotto Nocerino* PDO, a typical *Allium cepa* cultivar from Campania Region, Italy.

MAE platform was employed, leading to the valorisation of these residues, and enhancing the circular economy through improved waste management. For this purpose, BBD approach was effectively useful to maximize the extraction of TPC and FRAP from onion leaves.

Optimal MAE conditions to extract antioxidant compounds from CN leaves were determined using RSM (60 °C, 22 min, ethanol proportion of 51% (v/v), and solvent volume of 11 mL). These conditions provided a TPC value of 1.351 ± 0.07 mg GAE g^−1^ dw and an antioxidant capacity as measured by the FRAP assay of 14.016 ± 0.24 mmol Fe(II)E g^−1^ dw.

3D response curves showed that a moderate increase in ethanol concentration and higher extraction volume, coupled with extended extraction time and lower temperature, led to an enhanced yield of phenolic compounds and antioxidant activity in the final extracts.

A total of 63 compounds from various classes, including flavonoids, saponins, fatty acids, and lipids, were tentatively identified in the optimal CN extract using UHPLC-ESI-HR-MS/MS. Furthermore, we assessed the antioxidant potential of the CN extract on Hep G2 cells treated with H_2_O_2_. The results demonstrated a significant concentration-dependent inhibition of ROS release.

In conclusion, our study highlighted that spring onion leaves, often overlooked as agricultural by-products, are indeed a valuable source of antioxidant compounds. They could be used as functional ingredients for value-added products like functional foods and dietary supplements, thus providing innovative solutions for health and nutrition, while also contributing to the mitigation of environmental issues.

### Supplementary Information

Supplementary Information.

## Data Availability

The data and materials for this study are available from the corresponding author upon reasonable request.

## Abbreviations

ANOVA: Analysis of variance
BBD: Box–Behnken design
CN: *Cipollotto Nocerino*
DCFH-DA: 6-Carboxy-2ʹ,7ʹ-dichlorodihydrofluorescein diacetate
DHEX: Deoxyhexosyl
DPPH: 2,2-Diphenyl-1-picrylhydrazyl
ESI: Electrospray ionization
FA: Formic acid
FRAP: Ferric reducing antioxidant power
GAE: Gallic acid equivalents
HEX: Hexosyl
HRMS: High-resolution mass spectrometry
LC: Liquid chromatography
MAE: Microwave-assisted extraction
MS/MS: Tandem Mass Spectrometry
MTT: 3-[4,5-Dimethylthiazol-2,5-diphenyl-2H-tetrazolium bromide
OSW: Onion solid wastes
PEN: Pentosyl
ROS: Reactive oxygen species
RSM: Response surface methodology
SD: Standard deviation
TIC: Total ion chromatogram
TPC: Total phenolic content
TPTZ: 2,4,6-Trippyridyl-s-triazine
UAE: Ultrasound-assisted extraction
UHPLC: Ultra-high-performance liquid chromatography

## References

[1] Papaioannou EH, Mazzei R, Bazzarelli F, Piacentini E, Giannakopoulos V, Roberts MR, Giorno L (2022). Agri-food industry waste as resource of chemicals: The role of membrane technology in their sustainable recycling. Sustainability.

[2] Osorio LLDR, Flórez-López E, Grande-Tovar CD (2021). The potential of selected agri-food loss and waste to contribute to a circular economy: Applications in the food, cosmetic and pharmaceutical industries. Molecules.

[3] Fang B, Yu J, Chen Z, Osman AI, Farghali M, Ihara I, Hamza EH, Rooney DW, Yap PS (2023). Artificial intelligence for waste management in smart cities: A review. Environ. Chem. Lett..

[4] Chen T, Zhang S, Yuan Z (2020). Adoption of solid organic waste composting products: A critical review. J. Clean. Prod..

[5] Zaccardelli M, Pane C, Di Mola I, Ronga D, Mori M (2021). Municipal organic waste compost replaces mineral fertilization in the horticultural cropping systems, reducing the pollution risk. Ital. J. Agron..

[6] Srivastava V, De Araujo ASF, Vaish B, Bartelt-Hunt S, Singh P, Singh RP (2016). Biological response of using municipal solid waste compost in agriculture as fertilizer supplement. Rev. Environ. Sci. Bio/Technol..

[7] Cattaneo A, Federighi G, Vaz S (2021). The environmental impact of reducing food loss and waste: A critical assessment. Food Policy.

[8] Nile A, Nile SH, Kim DH, Keum YS, Seok PG, Sharma K (2018). Valorization of onion solid waste and their flavonols for assessment of cytotoxicity, enzyme inhibitory and antioxidant activities. Food Chem. Toxicol..

[9] Črnivec IGO, Skrt M, Šeremet D, Sterniša M, Farčnik D, Štrumbelj E, Poljanšek A, Cebin N, Pogačnik L, Možina SS (2021). Waste streams in onion production: Bioactive compounds, quercetin and use of antimicrobial and antioxidative properties. Waste Manag..

[10] Nile SH, Nile AS, Keum YS, Sharma K (2017). Utilization of quercetin and quercetin glycosides from onion (*Allium cepa* L.) solid waste as an antioxidant, urease and xanthine oxidase inhibitors. Food Chem..

[11] González-de-Peredo AV, Vázquez-Espinosa M, Espada-Bellido E, Ferreiro-González M, Carrera C, Barbero GF, Palma M (2022). Extraction of antioxidant compounds from onion bulb (*Allium cepa* L.) Using individual and simultaneous microwave-assisted extraction methods. Antioxidants.

[12] González-de-Peredo AV, Vázquez-Espinosa M, Espada-Bellido E, Ferreiro-González M, Carrera C, Barbero GF, Palma M (2021). Development of optimized ultrasound-assisted extraction methods for the recovery of total phenolic compounds and anthocyanins from onion bulbs. Antioxidants.

[13] Shang XC, Zhang YQ, Zheng YF, Yiqiang L (2022). Temperature-responsive deep eutectic solvents as eco-friendly and recyclable media for microwave extraction of flavonoid compounds from waste onion (*Allium cepa* L.) skins. Biomass Conv. Bioref..

[14] Jin EY, Lim S, Kim SO, Park Y-S, Jang JK, Chung M-S, Park H, Shim K-S, Choi YJ (2011). Optimization of various extraction methods for quercetin from onion skin using response surface methodology. Food Sci. Biotechnol..

[15] Viera VB, Piovesan N, Mello RDO, Barin JS, Fogaça ADO, Bizzi CA, Flores ÉMDM, Costa ACDS, Pereira DE, Soares JKB, Kubota EH (2021). Ultrasonic assisted extraction of phenolic compounds with evaluation of red onion skin (*Allium cepa* L.) antioxidant capacity. J. Culinary Sci. Technol..

[16] Benito-Román Ó, Blanco B, Sanz MT, Beltrán S (2021). Freeze-dried extract from onion (*Allium cepa* cv. Horcal) skin wastes: Extraction intensification and flavonoids identification. Food Bioprod. Process..

[17] Pal CBT, Jadeja GC (2019). Microwave-assisted deep eutectic solvent extraction of phenolic antioxidants from onion (*Allium cepa* L.) peel: A Box–Behnken design approach for optimization. J. Food Sci. Technol..

[18] Alves Filho EG, Lima M, Silva L, Ribeiro P, Tiwari BK, Fernandes FN, Brito ES (2021). Green ultrasound-assisted extraction of bioactive compounds from button mushrooms, potatoes, and onion peels. ACS Food Sci. Technol..

[19] Katsampa P, Valsamedou E, Grigorakis S, Makris DP (2015). A green ultrasound-assisted extraction process for the recovery of antioxidant polyphenols and pigments from onion solid wastes using Box–Behnken experimental design and kinetics. Ind. Crops Prod..

[20] Jang M, Asnin L, Nile SH, Keum YS, Kim HY, Park SW (2013). Ultrasound-assisted extraction of quercetin from onion solid wastes. Int. J. Food Sci. Technol..

[21] El-Hadidy EM, Mossa ME, Habashy HN (2014). Effect of freezing on the pungency and antioxidants activity in leaves and bulbs of green onion in Giza 6 and Photon varieties. Ann. Agric. Sci..

[22] Yuasa M, Akao Y, Kawabeta K, Tominaga M (2018). Antioxidant activity and characterization of taste in early fresh onions and their leaves produced in Minamishimabara, Nagasaki, Japan. J. Home Econ. Jpn..

[23] Commission Regulation (EC) (2008). No 656/2008 registering certain names in the Register of protected designations of origin and protected geographical indications (*Chamomilla Bohemica* (PDO), *Vlaams-Brabantse tafeldruif* (PDO), *Slovenská parenica* (PGI), *Cipollotto Nocerino* (PDO)). Off. J. Eur. Commun..

[24] Dabeek WM, Marra MV (2019). Dietary quercetin and kaempferol: Bioavailability and potential cardiovascular-related bioactivity in humans. Nutrients.

[25] Aoyama S, Yamamoto Y (2007). Antioxidant activity and flavonoid content of Welsh onion (*Allium fistulosum*) and the effect of thermal treatment. Food Sci. Technol. Res..

[26] Issa M, Karabet F, Aljoubbeh M (2013). Total polyphenols, flavonoid content, kaempferol concentration and antioxidant activity of two onion Syrian (spring and white). Int. J. ChemTech. Res..

[27] Chang T-C, Jang H-D, Lin W-D, Duan P-F (2016). Antifungal activities of commercial rice wine extracts of Taiwanese *Allium fistulosum*. Adv. Microbiol..

[28] Karabegović I, Nikolova M, Veličković D, Stojičević S, Veljković V, Lazić M (2011). Comparison of antioxidant and antimicrobial activities of methanolic extracts of the *Artemisia* sp. recovered by different extraction techniques. Chin. J. Chem. Eng..

[29] Štajner D, Milić N, Čanadanović-Brunet J, Kapor A, Štajner M, Popović B (2006). Exploring Allium species as a source of potential medicinal agents. Phytother. Res..

[30] Kurnia D, Ajiati D, Heliawati L, Sumiarsa D (2021). Antioxidant properties and structure-antioxidant activity relationship of Allium species leaves. Molecules.

[31] Medina-Jaramillo C, Gomez-Delgado E, López-Córdoba A (2022). Improvement of the ultrasound-assisted extraction of polyphenols from welsh onion (*Allium fistulosum*) leaves using response surface methodology. Foods.

[32] Senrayan J, Venkatachalam S (2019). Optimization of ultrasound-assisted solvent extraction (UASE) based on oil yield, antioxidant activity and evaluation of fatty acid composition and thermal stability of *Coriandrum sativum* L. seed oil. Food Sci. Biotechnol..

[33] Hossain MB (2012). Optimization of ultrasound assisted extraction of antioxidant compounds from marjoram (*Origanum majorana* L.) using response surface methodology. Ultrason. Sonochem..

[34] Vinatoru M, Mason T, Calinescu I (2017). Ultrasonically assisted extraction (UAE) and microwave assisted extraction (MAE) of functional compounds from plant materials. Trends Anal. Chem..

[35] Grigonis D, Venskutonis P, Sivik B, Sandahl M, Eskilsson CS (2005). Comparison of different extraction techniques for isolation of antioxidants from sweet grass (*Hierochloe odorata*). J. Supercrit. Fluids.

[36] De La Hoz A, Alcázar J, Carrillo J, Herrero MA, Muñoz JDM, Prieto P, De Cózar A, Diaz-Ortiz A, Chandra U (2011). Reproducibility and scalability of microwave-assisted reactions. Microwave Heating.

[37] Li Y, Radoiu M, Fabiano-Tixier AS, Chemat F, Chemat F, Cravotto G (2013). From laboratory to industry: Scale-up, quality, and safety consideration for microwave-assisted extraction. Microwave-Assisted Extraction for Bioactive Compounds. Food Engineering Series.

[38] Chan CH, Yusoff R, Ngoh GC (2015). Assessment of scale-up parameters of microwave-assisted extraction via the extraction of flavonoids from cocoa leaves. Chem. Eng. Technol..

[39] Way ML, Jones JE, Nichols DS, Dambergs RG, Swarts ND (2020). A comparison of laboratory analysis methods for total phenolic content of cider. Beverages.

[40] Noreen H, Semmar N, Farman M, McCullagh JS (2017). Measurement of total phenolic content and antioxidant activity of aerial parts of medicinal plant *Coronopus didymus*. Asian Pac J Trop Med.

[41] Vestuto V, Amodio G, Pepe G, Basilicata MG, Belvedere R, Napolitano E, Guarnieri D, Pagliara V, Paladino S, Rodriquez M (2022). Cocoa extract provides protection against 6-OHDA toxicity in SH-SY5Y dopaminergic neurons by targeting PERK. Biomedicines.

[42] Guo Z, Jin Q, Fan G, Duan Y, Qin C, Wen M (2001). Microwave-assisted extraction of effective constituents from a Chinese herbal medicine *Radix puerariae*. Anal. Chim. Acta.

[43] Imtiaz F, Ahmed D, Abdullah RH, Ihsan S (2023). Green extraction of bioactive compounds from *Thuja orientalis* leaves using microwave- and ultrasound-assisted extraction and optimization by response surface methodology. Sustain. Chem. Pharm..

[44] Bouras M, Chadni M, Barba FJ, Grimi N, Bals O, Vorobiev E (2015). Optimization of microwave-assisted extraction of polyphenols from *Quercus* bark. Ind. Crops Prod..

[45] Zhang H, Li H, Zhang Z, Hou T (2021). Optimization of ultrasound-assisted extraction of polysaccharides from perilla seed meal by response surface methodology: Characterization and in vitro antioxidant activities. J. Food Sci..

[46] Náthia-Neves G, Alonso E (2021). Valorization of sunflower by-product using microwave-assisted extraction to obtain a rich protein flour: Recovery of chlorogenic acid, phenolic content and antioxidant capacity. Food Bioprod. Process..

[47] Tatke P, Jaiswal Y (2011). An overview of microwave assisted extraction and its applications in herbal drug research. Res. J. Med. Plants.

[48] Xiaokang W, Lyng JG, Brunton NP, Cody L, Jacquier JC, Harrison SM, Papoutsis K (2020). Monitoring the effect of different microwave extraction parameters on the recovery of polyphenols from shiitake mushrooms: Comparison with hot-water and organic-solvent extractions. Biotechnol. Rep..

[49] Lovrić V, Putnik P, Bursać Kovačević D, Jukić M, Dragović-Uzelac V (2017). Effect of microwave-assisted extraction on the phenolic compounds and antioxidant capacity of blackthorn flowers. Food Bioprod. Process..

[50] Da Rocha CB, Noreña CPZ (2020). Microwave-assisted extraction and ultrasound-assisted extraction of bioactive compounds from grape pomace. Int. J. Food Eng..

[51] Chuyen HV, Nguyen MH, Roach PD, Golding JB, Parks SE (2017). Microwave-assisted extraction and ultrasound-assisted extraction for recovering carotenoids from Gac peel and their effects on antioxidant capacity of the extracts. Food Sci. Nutr..

[52] Yang Y-C, Li J, Zu Y-G, Fu Y-J, Luo M, Wu N, Liu X-L (2010). Optimisation of microwave-assisted enzymatic extraction of corilagin and geraniin from *Geranium sibiricum* Linne and evaluation of antioxidant activity. Food Chem..

[53] Dahmoune F, Nayak B, Moussi K, Remini H, Madani K (2015). Optimization of microwave-assisted extraction of polyphenols from *Myrtus communis* L. leaves. Food Chem..

[54] Garcia-Castello EM, Rodriguez-Lopez AD, Mayor L, Ballesteros R, Conidi C, Cassano A (2015). Optimization of conventional and ultrasound assisted extraction of flavonoids from grapefruit (*Citrus paradisi* L.) solid wastes. LWT Food Sci. Technol..

[55] Ghafoor K, Choi YH, Jeon JY, Jo IH (2009). Optimization of ultrasound-assisted extraction of phenolic compounds, antioxidants, and anthocyanins from grape (*Vitis vinifera*) seeds. J. Agric. Food Chem..

[56] Sridhar A, Ponnuchamy M, Kumar PS, Kapoor A, Vo D-VN, Prabhakar S (2021). Techniques and modeling of polyphenol extraction from food: A review. Environ. Chem. Lett..

[57] Tedesco I, Carbone V, Spagnuolo C, Minasi P, Russo GL (2015). Identification and quantification of flavonoids from two southern Italian cultivars of *Allium cepa* L., Tropea (Red Onion) and Montoro (Copper Onion), and their capacity to protect human erythrocytes from oxidative stress. J. Agric. Food Chem..

[58] Dabeek WM, Kovinich N, Walsh C, Ventura Marra M (2019). Characterization and quantification of major flavonol glycosides in ramps (*Allium tricoccum*). Molecules.

[59] Kumar A, Singh N, Kaur A, Joshi R (2023). Sneak-peek into the chlorophyll content, antioxidant activity, targeted and non-targeted UHPLC-QTOF LC/MS metabolomic fingerprints of pulse microgreens grown under different photoperiod regimes. Food Biosci..

[60] Yang D, Dunshea FR, Suleria HA (2020). LC-ESI-QTOF/MS characterization of Australian herb and spices (garlic, ginger, and onion) and potential antioxidant activity. J. Food Process. Preserv..

[61] Danise T, Innangi M, Curcio E, Piccolella S, Fioretto A, Pacifico S (2021). White poplar (*Populus alba* L.) leaf waste recovery and intercropping outcome on its polyphenols. Ind. Crops Prod..

[62] Farag MA, Ali SE, Hodaya RH, El-Seedi HR, Sultani HN, Laub A, Eissa TF, Abou-Zaid FO, Wessjohann LA (2017). Phytochemical profiles and antimicrobial activities of *Allium cepa* red cv. and *A. sativum* subjected to different drying methods: A comparative MS-based metabolomics. Molecules.

[63] Schmidt S, Zietz M, Schreiner M, Rohn S, Kroh LW, Krumbein A (2010). Identification of complex, naturally occurring flavonoid glycosides in kale (*Brassica oleracea* var. *sabellica*) by high-performance liquid chromatography diode-array detection/electrospray ionization multi-stage mass spectrometry. Rapid Commun. Mass Spectrom..

[64] Zhou Y, Li C, Feng B, Chen B, Jin L, Shen Y (2020). UPLC-ESI-MS/MS based identification and antioxidant, antibacterial, cytotoxic activities of aqueous extracts from storey onion (*Allium cepa* L. var. *proliferum* Regel). Food Res. Int..

[65] Geng P, Chen P, Lin L-Z, Sun J, Harrington P, Harnly JM (2021). Classification of structural characteristics facilitate identifying steroidal saponins in Alliums using ultra-high performance liquid chromatography high-resolution mass spectrometry. J. Food Compos. Anal..

[66] Maryuni DR, Prameswari DA, Astari SD, Sari SP, Putri DN (2022). Identification of active compounds in red onion (*Allium ascalonicum* L.) peel extract by Lc-Esi-Qtof-Ms/Ms and determination of its antioxidant activity. J. Teknol. Hasil Pertan..

[67] FooDB. *FooDB Version 1.0* (2020).

[68] Park SK, Ha JS, Kim JM, Kang JY, Lee DS, Guo TJ, Lee U, Kim D-O, Heo HJ (2016). Antiamnesic effect of broccoli (*Brassica oleracea* var. *italica*) leaves on amyloid beta (Aβ) 1–42-induced learning and memory impairment. J. Agric. Food Chem..

[69] CompoundDiscoverer. Compound Discoverer v. 3.3.1.111 SP1. In *Thermo Fisher Scientific, Bremen, Germany*.

[70] Bontempo P, Stiuso P, Lama S, Napolitano A, Piacente S, Altucci L, Molinari AM, De Masi L, Rigano D (2021). Metabolite profile and in vitro beneficial effects of black garlic (*Allium sativum* L.) polar extract. Nutrients.

[71] González-Peña D, Dudzik D, García A, de Ancos B, Barbas C, Sánchez-Moreno C (2017). Metabolomic fingerprinting in the comprehensive study of liver changes associated with onion supplementation in hypercholesterolemic Wistar rats. Int. J. Mol. Sci..

[72] Liebisch G, Fahy E, Aoki J, Dennis EA, Durand T, Ejsing CS, Fedorova M, Feussner I, Griffiths WJ, Köfeler H (2020). Update on LIPID MAPS classification, nomenclature, and shorthand notation for MS-derived lipid structures. J. Lipid Res..

[73] Tsiaganis MC, Laskari K, Melissari E (2006). Fatty acid composition of Allium species lipids. J. Food Compos. Anal..

[74] Song H, Wu H, Geng Z, Sun C, Ren S, Wang D, Zhang M, Liu F, Xu W (2016). Simultaneous determination of 13-HODE, 9, 10-DHODE, and 9, 10, 13-THODE in cured meat products by LC-MS/MS. Food Anal. Methods.

[75] Chen K, Ríos JJ, Pérez-Gálvez A, Roca M (2017). Comprehensive chlorophyll composition in the main edible seaweeds. Food Chem..

[76] Foti MC (2015). Use and abuse of the DPPH• radical. J. Agric. Food Chem..

[77] Benzie IF, Strain JJ (1996). The ferric reducing ability of plasma (FRAP) as a measure of “antioxidant power”: The FRAP assay. Anal. Biochem..

[78] Pasqualetti V, Locato V, Fanali C, Mulinacci N, Cimini S, Morgia AM, Pasqua G, De Gara L (2021). Comparison between in vitro chemical and ex vivo biological assays to evaluate antioxidant capacity of botanical extracts. Antioxidants.

[79] Pepe G, Basilicata MG, Carrizzo A, Adesso S, Ostacolo C, Sala M, Sommella E, Ruocco M, Cascioferro S, Ambrosio M, Pisanti S, Di Sarno V, Bertamino A, Marzocco S, Vecchione C, Campiglia P (2019). β-Lactoglobulin heptapeptide reduces oxidative stress in intestinal epithelial cells and angiotensin II-induced vasoconstriction on mouse mesenteric arteries by induction of nuclear factor erythroid 2-related factor 2 (Nrf2) translocation. Oxid. Med. Cell. Longev..

[80] Arzumanian VA, Kiseleva OI, Poverennaya EV (2021). The curious case of the HepG2 cell line: 40 years of expertise. Int. J. Mol. Sci..

[81] Martins GR, Mattos MMG, Nascimento FM, Brum FL, Mohana-Borges R, Figueiredo NG (2022). Phenolic profile and antioxidant properties in extracts of developing Açaí (*Euterpe oleracea* Mart.) seeds. J. Agric. Food Chem..

