# Supplementary material for: Optimization of microwave-assisted extraction of antioxidant compounds from spring onion leaves using Box–Behnken design
Source: Sci Rep. 2023 Sep 10;13:14923. doi: 10.1038/s41598-023-42303-x (PMC10493223; doi:10.1038/s41598-023-42303-x)
Supplement: Supplementary file 1 — Supplementary Information. [file 41598_2023_42303_MOESM1_ESM.pdf]

Supporting Informations

Optimization of Microwave-Assisted Extraction of Antioxidant Compounds from Spring Onion Leaves using Box-Behnken Design

Giovanna Aquino[1,2], Manuela Giovanna Basilicata[1,*], Carlo Crescenzi[1], Vincenzo Vestuto[1], Emanuela Salviati[1], Michele Cerrato[1], Tania Ciaglia[1], Francesca Sansone[1], Giacomo Pepe [1], Pietro Campiglia [1]

[1] Department of Pharmacy, University of Salerno, 84084 Fisciano, SA, Italy

[2] PhD Program in Drug Discovery and Development, University of Salerno, Fisciano, SA, Italy

Corresponding Authors: Manuela Giovanna Basilicata (mbasilicata@unisa.it)

Contents:

Table S1. Extraction Yield (%) and DPPH assay values.[80]

Table S2. Analysis of variance (ANOVA) for DPPH radical scavenging activity of CN extracts

Table S3 Systematic classification of Allium sapogenins and substituents in related steroidal saponins.

Figure S1. Representative TIC of CN extract analyzed in negative (up) and positive (down) ionization mode.

Table S1. Extraction Yield (%) and DPPH assay values.[80]

Run	Factors				Yield (%) *	DPPH*# (μM TE g^{-1} dw)
	A-Temp	B-Time	C-Extr. Vol.	D-EtOH		
**1**	-1	-1	0	0	26.97 ± 1.46	19.04 ± 0.90
**2**	0	-1	-1	0	22.93 ± 1.23	21.60 ± 0.54
**3**	0	-1	0	-1	22.10 ± 0.79	22.57 ± 0.88
**4**	0	-1	0	+1	23.30 ± 0.30	22.97 ± 0.38
**5**	0	-1	+1	0	14.83 ± 1.00	22.61 ± 0.26
**6**	+1	-1	0	0	19.17 ± 0.26	23.03 ± 0.46
**7**	-1	0	-1	0	19.37 ± 1.20	20.45 ± 0.65
**8**	-1	0	0	-1	28.63 ± 1.00	20.31 ± 0.49
**9**	-1	0	0	+1	25.10 ± 1.05	21.91 ± 0.37
**10**	-1	0	+1	0	20.93 ± 1.52	23.24 ± 0.44
**11**	0	0	-1	-1	26.60 ± 0.26	19.32 ± 0.08
**12**	0	0	-1	+1	21.57 ± 0.70	20.41 ± 0.37
**13**	0	0	0	0	23.87 ± 1.60	17.67 ± 0.03
**14**	0	0	0	0	20.57 ± 0.75	22.47 ± 3.34
**15**	0	0	0	0	29.20 ± 0.26	20.73 ± 0.97
**16**	0	0	0	0	22.80 ± 1.04	23.90 ± 0.34
**17**	0	0	0	0	22.63 ± 1.30	20.30 ± 0.30
**18**	0	0	+1	-1	16.63 ± 1.56	21.42 ± 1.88
**19**	0	0	+1	+1	27.50 ± 1.40	21.03 ± 0.20
**20**	+1	0	-1	0	20.47 ± 1.28	22.12 ± 0.27
**21**	+1	0	0	-1	25.20 ± 0.36	20.95 ± 0.11
**22**	+1	0	0	+1	22.67 ± 1.53	24.51 ± 1.82
**23**	+1	0	+1	0	20.73 ± 1.45	17.28 ± 1.42
**24**	-1	+1	0	0	24.77 ± 1.55	21.40 ± 0.23
**25**	0	+1	-1	0	24.40 ± 1.06	20.76 ± 0.23
**26**	0	+1	0	-1	22.67 ± 0.64	21.26 ± 0.29
**27**	0	+1	0	+1	20.20 ± 1.47	21.61 ± 0.13
**28**	0	+1	+1	0	21.40 ± 1.20	21.67 ± 0.14
**29**	+1	+1	0	0	23.30 ± 0.66	21.15 ± 0.24
**Optimal extract**	60°C	22 in	11 mL	51 v/v	22.90 ± 0.76	22.85 ± 0.15

** Mean ± SD (n=3)*

The results were expressed as μM Trolox equivalents/g dried extract.

Table S2. Analysis of variance (ANOVA) for DPPH radical scavenging activity of CN extracts.

Source	Sum of Squares	df	Mean Square	F-value	*p*-value
**Model**	69.58	14	4.97	11.97	< 0.0001*
A-Temp.	4.80	1	4.80	11.56	0.0043*
B-Time	35.19	1	35.19	84.77	< 0.0001*
C-Extr. Vol.	3.20	1	3.20	7.72	0.0148*
D-EtOH	24.31	1	24.31	58.56	< 0.0001*
AB	1.17	1	1.17	2.81	0.1159
AC	0.0002	1	0.0002	0.0005	0.9818
AD	0.0144	1	0.0144	0.0347	0.8549
BC	0.6642	1	0.6642	1.60	0.2266
BD	0.0784	1	0.0784	0.1888	0.6705
CD	0.0784	1	0.0784	0.1888	0.6705
A^2	0.0129	1	0.0129	0.0311	0.8626
B^2	0.0237	1	0.0237	0.0570	0.8147
C^2	0.0221	1	0.0221	0.0532	0.8210
D^2	0.0024	1	0.0024	0.0057	0.9407
**Residual**	5.81	14	0.4151		
Lack of Fit	5.26	10	0.5264	3.84	0.1033
Pure Error	0.5482	4	0.1371		
**R^2**	0.9229				
**Adjusted R^2**	0.8458				
**Predicted R^2**	0.5865				

**Significant at $p < 0.05$.*

Table S3. Systematic classification of Allium sapogenins and substituents in related steroidal saponins.

Class	Subclass	Formula of $[M-H]^-$ ion	$[M-H]^-$ (Da)	Allium Sapogenins
**III**	III3	$C_{27}H_{41}O_4$	429	(25R/S)-furost-5(6),20(22)-diene-2α,3β,26-triols; 5β-furost-20(22),25(27)-diene-3β,12β,26-triol; furost-5(6),20(22)-diene-1β,3β,26-triol<br>(25R)-5α-furost-20(22)-ene-6-one-3β,26-diol<br>(22S,25S)-22,25-epoxy-furost-5(6)-ene-3β,22,26-triol<br>(25R/S)-spirost-5(6)-ene-1β,3β-; 2α,3β-;3β,3β-diols; (25R)-spiros-25(27)-ene-2β,3β-;3β,12β-diols<br>(25S)-spirostan-6-one-3-OH (laxogenin); (25R)-5α-spirostane-12-one-3β-ol (hecogenin)
	III4	$C_{27}H_{41}O_5$	445	(22S, 25R)-5α-furost-5(6),22(23)-dien1β,3β,20α,26-tetrol<br>(25R)-furost-5(6)-ene-2-one-3β(α),22α(22),26-triol, (25R)-furost-25(27)-en-12-one3β,22,26-triol<br>(25R)-spirost-5(6)-ene1β,3β,24-triol, (20S,25S)-spirost-5(6)-ene-3β,11α(12β),21-tiols, (24S,25R)-spirostS(6)-ene-β(2α),3β,24-triol<br>(25R/S)-5α-spirostan-2-one-3β,6β-diol; (25S)-5α-spirostane-6-one2α,3α-diol; (25R)-5αspirostane-12-one3β,6β-diol<br>(25R)-5α-spirostane2α,5α-epoxy-2α,3β,6βtriol
**IV**	IV4	$C_{27}H_{43}O_5$	447	(25R/S)-furost-5(6)-ene-1β(2α),3β,22α(β),26-tetraols; (25S,20R)-5α-furost-22(23)-ene-2α,3β,20,26-tetrol; 5α(β)-furost-25(27)-ene-3β,12β,22,26-tetraol<br>(25R)-5α-furostane-6-one-3β,22,26-triol<br>(25R/S)-spirostan-2α (β),3β,6β(α); 2,3,27-; 2,3,24-; 2,3,5-; 3,5,6-triol; (24S,25S)-5β-spirostan-2β,3β,24-triol
**Neutral Loss**	**Abbreviations**	**Formula**	**Neutral Loss**	**Example Substituents**
**hexosyl**	Hex	$C_6H_{10}O_5$	162.0528	glucosyl, galactosyl, mannosyl
**deoxyhexosyl**	dHex	$C_6H_{10}O_4$	146.0579	rhamnosyl, deoxymannosyl, quinovosyl
**pentosyl**	Pen	$C_5H_8O_4$	132.0422	arabinosyl, xylosyl
**formic acid**	FA	CH_2O_2	46.0055	

Figure S1. Representative TIC of CN extract analyzed in negative (up) and positive (down) ionization mode. 1. Quercetin 7,4′- dihexoside; 2. Herniarin; 3. Kaempferol 3,7-O-dihexoside; 4. Kaempferol 3,7-O-dihexoside (isomer I); 5. Quercetin 3,4′- dihexoside; 6. Cyanidin 3-laminaribioside; 7. Quercetin 3,4′- dihexoside (isomer I); 8. Quercetin 3-O-hexoside; 9. Kaempferol; 10. Phenethyl rutinoside; 11. Quercetin; 12. Kaempferol 3,7-O-dihexoside (isomer II); 13. Kaempferol 3-O-hexoside; 14. Quercetin 3-O-hexoside (isomer I); 15. Kaempferol 3-O-hexoside (isomer I); 16. Isorhamnetin-O-hexoside; 17. Kaempferol (isomer I); 18. Quercetin-3-O-feruloyl-sophoroside-7-O-D-glucoside; 19. Petunidin 3-hexoside; 20. Quercetin-3-O-feruloyl-sophoroside-7-O-D-glucoside (isomer I); 21. Kaempferol-3-O-coumaroyldiglucoside-7-O-glucoside; 22. Isorhamnetin-O-hexoside (isomer I); 23. Kaempferol-3-O-feruloyldiglucoside-7-O-glucoside; 24. Kaempferol-3-O-coumaroyldiglucoside-7-O-glucoside (isomer I); 25. Kaempferol-3-O-feruloyldiglucoside-7-O-glucoside (isomer I); 26. Saponin 3-IV4-1 (447+dHex+2 Hex + FA); 27. Neohecogenin-3- Oβ-Dglucopyranosyl (1→2)-β-D-glucopyranosyl (1→4)-β-D-galactopy-ranoside; 28. Saponin 3-IV4-1 (isomer I) (447+ dHex+2 Hex + FA); 29. Neohecogenin-3- Oβ-Dglucopyranosyl (1→2)-β-D-glucopyranosyl (1→4)-β-D-galactopy-ranoside (isomer I); 30. 7-Hydroxy-2’,4’,5-trimethoxyflavanone; 31. Saponin 3-IV4-2 (447+Pen + dHex +Hex + FA); 32. Pennogenin-3-O-α-L-arabinofuranosyl(1→4)[α-L-rhamnopyranosyl(1→2)]-β-D-glucopyranoside; 33. Quercetin-3-O-feruloyl-sophoroside; 34. Kaempferol (isomer II); 35. Oxo-dihydroxy-octadecenoic acid (oxoDiHODE); 36. 9,12,13-trihydroxy octadeca-7-enoic acid (TriHODE); 37. 9,12,13-trihydroxy octadeca-7-enoic acid (TriHODE) (isomer II); 38. Saponin 2-III4 (445+dHex + Pen + FA); 39. 9,12,13-trihydroxy octadeca-7-enoic acid (TriHODE) (isomer III); 40. Palmitoylglycine; 41. Palmitoylglycine (isomer I); 42. 2'-Hydroxy-4,4',6'-trimethoxychalcone; 43. 5,6,7,4'-Tetramethoxyflavanone; 44. Dehydrophytosphingosine; 45. Palmitoylglycine (isomer II); 46. Dehydrophytosphingosine (isomer I); 47. Phytosphingosine; 48. Tigogenin; 49. Saponin 2-III3 (429+dHex + Pen + FA); 50. Hydroxyoctadecatrienoic acid (HOTrE); 51. LysoPC(16:0); 52. 13-hydroxyoctadecadienoic acid (13-HODE); 53. 13-hydroxyoctadecadienoic acid (isomer I); 54. α-Linolenoyl ethanolamide; 55. Linoleoyl ethanolamide; 56. Hydroxy-hexadecanoic acid; 57. 3-dehydrosphinganine (C20); 58. Hexadecanamide; 59. Sphingosine ; 60. Pheophorbide a; 61. Octadecanamide; 62. 1,3-dilinolenoylglycerol (DG(18:3n6/0:0/18:3n6)); 63. 1,3-dilinolenoylglycerol (DG(18:3n6/0:0/18:3n6)) (isomer I)

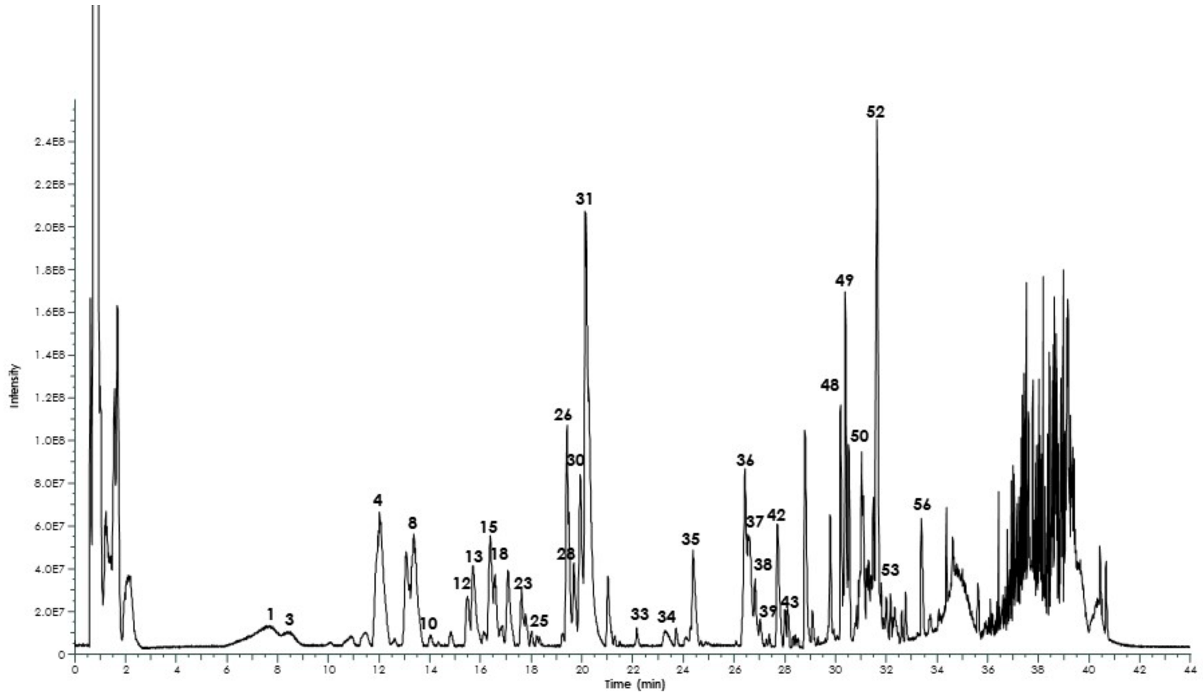

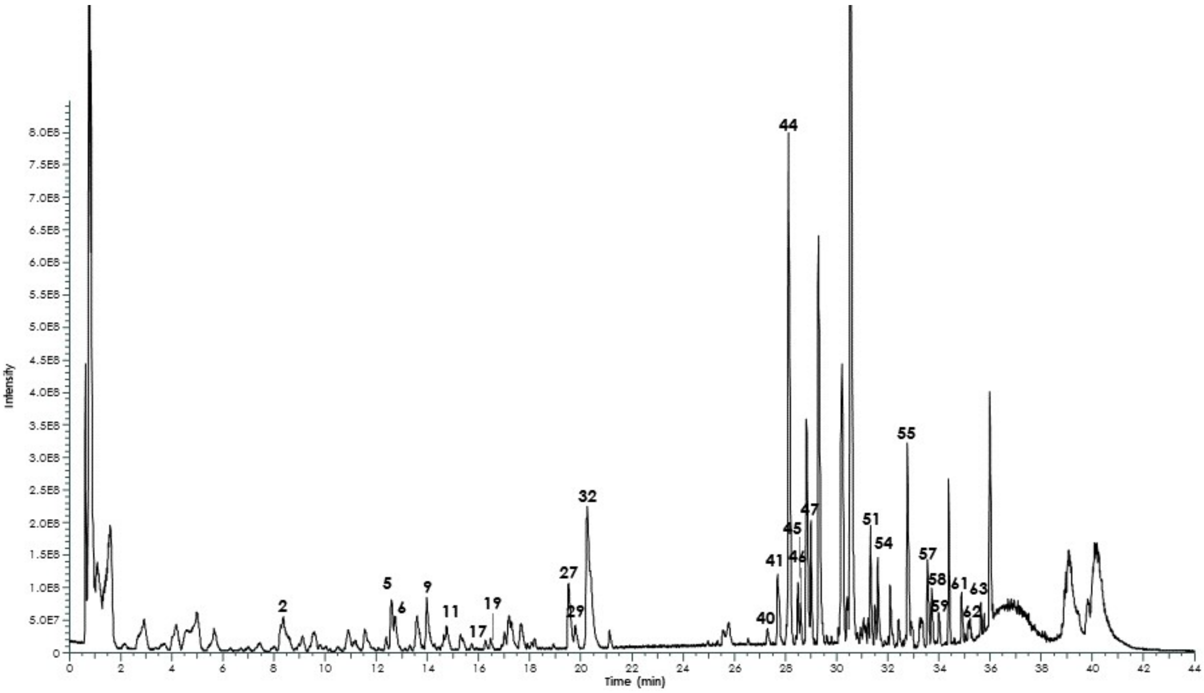
Intensity
8.0E8
7.5E8
7.0E8
6.5E8
6.0E8
5.5E8
5.0E8
4.5E8
4.0E8
3.5E8
3.0E8
2.5E8
2.0E8
1.5E8
1.0E8
5.0E7
0
0 2 4 6 8 10 12 14 16 18 20 22 24 26 28 30 32 34 36 38 40 42 44
Time (min)
2
5
6
9
11
17
19
27
29
32
40
41
44
45
46
47
51
54
55
57
58
59
61
62
63
